# Spatial risk analysis for the introduction and circulation of six arboviruses in the Netherlands

**DOI:** 10.1186/s13071-020-04339-0

**Published:** 2020-09-10

**Authors:** Helen Joan Esser, Yorick Liefting, Adolfo Ibáñez-Justicia, Henk van der Jeugd, Chris A. M. van Turnhout, Arjan Stroo, Chantal B. E. M. Reusken, Marion P. G. Koopmans, Willem Fred de Boer

**Affiliations:** 1 Wildlife Ecology & Conservation Group, Wageningen University & Research, Wageningen, The Netherlands; 2 Laboratory of Entomology, Wageningen University & Research, Wageningen, The Netherlands; 3 Centre for Infectious Disease Control, National Institute for Public Health and the Environment, Bilthoven, The Netherlands; 4 Centre for Monitoring of Vectors (CMV), National Reference Centre (NRC), Netherlands Food and Consumer Product Safety Authority (NVWA), Ministry of Agriculture, Nature and Food Quality, Wageningen, The Netherlands; 5 Vogeltrekstation - Dutch Centre for Avian Migration and Demography (NIOO-KNAW), Wageningen, The Netherlands; 6 Sovon Dutch Centre for Field Ornithology, Nijmegen, The Netherlands; 7 Department of Animal Ecology & Ecophysiology, Institute for Water and Wetland Research, Radboud University, Nijmegen, The Netherlands; 8 Department of Viroscience, WHO CC for Arbovirus and Viral Hemorrhagic Fever Reference and Research, Erasmus University Medical Centre, Rotterdam, The Netherlands

**Keywords:** Risk mapping, Geographic Information System, West Nile virus, Japanese encephalitis virus, Rift Valley fever virus, Tick-borne encephalitis virus, Louping-ill virus, Crimean-Congo haemorrhagic fever virus, Vector-borne diseases

## Abstract

**Background:**

Arboviruses are a growing public health concern in Europe, with both endemic and exotic arboviruses expected to spread further into novel areas in the next decades. Predicting where future outbreaks will occur is a major challenge, particularly for regions where these arboviruses are not endemic. Spatial modelling of ecological risk factors for arbovirus circulation can help identify areas of potential emergence. Moreover, combining hazard maps of different arboviruses may facilitate a cost-efficient, targeted multiplex-surveillance strategy in areas where virus transmission is most likely. Here, we developed predictive hazard maps for the introduction and/or establishment of six arboviruses that were previously prioritized for the Netherlands: West Nile virus, Japanese encephalitis virus, Rift Valley fever virus, tick-borne encephalitis virus, louping-ill virus and Crimean-Congo haemorrhagic fever virus.

**Methods:**

Our spatial model included ecological risk factors that were identified as relevant for these arboviruses by an earlier systematic review, including abiotic conditions, vector abundance, and host availability. We used geographic information system (GIS)-based tools and geostatistical analyses to model spatially continuous datasets on these risk factors to identify regions in the Netherlands with suitable ecological conditions for arbovirus introduction and establishment.

**Results:**

The resulting hazard maps show that there is spatial clustering of areas with either a relatively low or relatively high environmental suitability for arbovirus circulation. Moreover, there was some overlap in high-hazard areas for virus introduction and/or establishment, particularly in the southern part of the country.

**Conclusions:**

The similarities in environmental suitability for some of the arboviruses provide opportunities for targeted sampling of vectors and/or sentinel hosts in these potential hotspots of emergence, thereby increasing the efficient use of limited resources for surveillance.
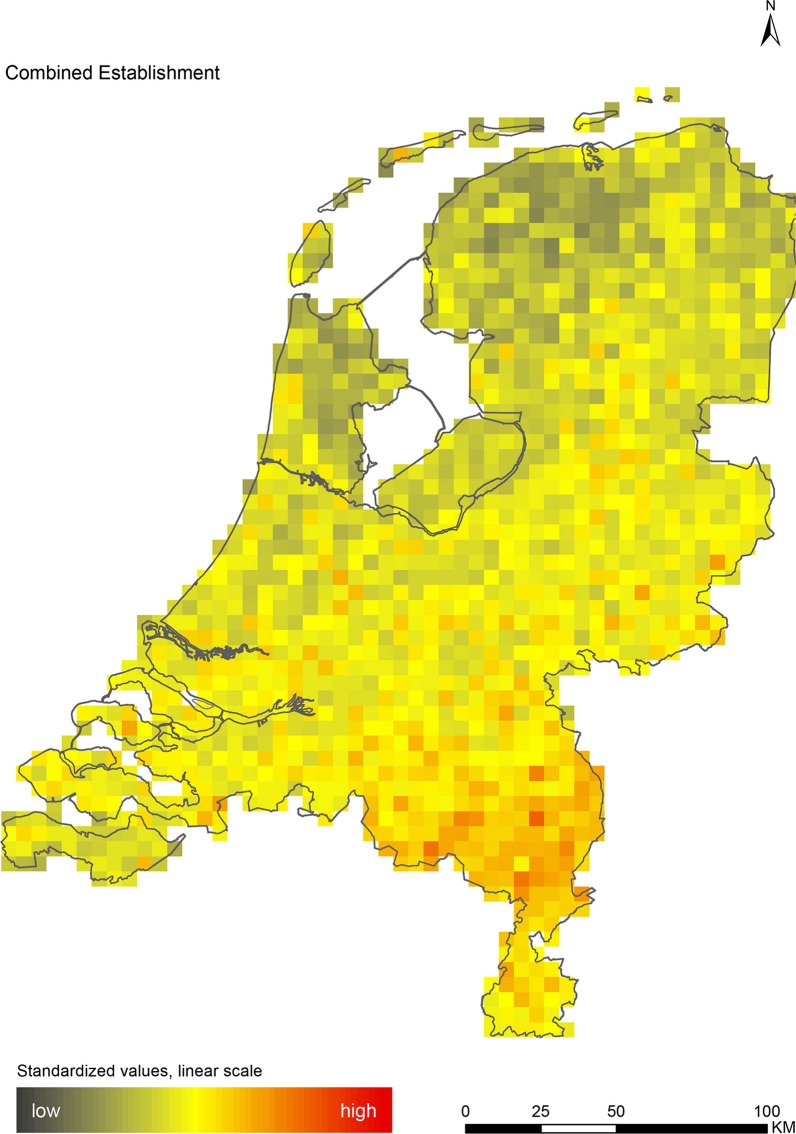

## Background

Arthropod-borne viruses, or arboviruses, are an increasing public health concern in Europe [1]. Diseases caused by endemic arboviruses such as West Nile virus (WNV), Crimean-Congo haemorrhagic fever virus (CCHFV), and tick-borne encephalitis virus (TBEV) are all increasing in incidence and distribution [2–4]. Moreover, the widespread occurrence and periodically high local abundance of competent vectors and reservoir hosts increase the probability that exotic arboviruses, such as Japanese encephalitis virus (JEV) or Rift Valley fever virus (RVFV), become established [5, 6]. Projected changes in land-use and climate, socio-economic development, and virus evolution may all contribute to larger and more frequent outbreaks in endemic regions, and promote geographic expansion of arboviruses, including those that were traditionally confined to tropical regions, into novel areas within Europe [7–10].

The above-mentioned mosquito-borne viruses (JEV, WNV and RVFV) and tick-borne viruses (CCHFV, TBEV and the closely related louping-ill virus LIV) have been marked as top priority arboviruses for the Netherlands based on epidemiological criteria, and their economic and societal impact [11]. Their potential emergence in the Netherlands may be facilitated by the country’s unique combination of (i) high densities of livestock, which function as reservoir hosts for JEV, RVFV, CCHFV and LIV, (ii) great global connectivity in trade and travel through large airports and seaports, which increases the risk for arbovirus introduction, and (iii) priority policy to improve ecological conditions attracting wildlife (both resident and migratory) *via* habitat conservation and the establishment of wildlife corridors. Examples of these conservation initiatives are the National Ecological Network (NEN), the Natura 2000 network, and the Pan-European Ecological Network (PEEN), which are all aimed at higher wildlife mobility and larger distribution ranges, and hence may facilitate the spread of arboviruses with wildlife reservoirs. Moreover, increasingly warmer summers and milder winters improve the climatic suitability of the Netherlands for the establishment of arboviruses and their vectors [12, 13]. The recent outbreaks of Usutu virus in blackbirds and the first autochthonous human cases of TBE in this country, underline the continuous threat of arbovirus emergence [14, 15].

While history has shown that preventing the introduction and spread of arboviruses and their vectors into novel areas may be near impossible [16–19], potential outbreaks can be prevented or their effects mitigated by targeted early warning surveillance, preparedness planning, and control efforts (e.g. monitoring of vectors and/or sentinel hosts, vaccination, education campaigns and biological control) in hazardous areas [20, 21]. However, predicting where arbovirus emergence is most likely to occur and hence, selecting locations where surveillance should be most effective, is a major challenge. A spatial analysis of ecological risk factors for arbovirus circulation can help identify such areas. Arboviruses are associated with the presence, abundance and interactions of specific vectors, reservoir hosts, and abiotic conditions that need to converge for arbovirus replication and transmission to occur [22]. Spatial modelling of these ecological risk factors can be used to map the environmental suitability (‘hazard’) for local circulation of arboviruses in regions beyond their current distribution [23–27].

In this study, we developed predictive hazard maps for the introduction and/or establishment of the six earlier prioritized arboviruses TBEV, LIV, CCHFV, JEV, WNV and RVFV in the Netherlands. Our spatial analysis included ecological risk factors that were identified as relevant for these arboviruses by an earlier systematic review, including abiotic conditions, vector abundance, and host availability [28]. We used geographic information system (GIS)-based tools and geostatistical analyses to model spatially continuous data on these risk factors to identify regions with suitable ecological conditions for endemic circulation. It is in these potential hotspots where surveillance efforts should be focused to enable early risk management. Our specific goal was to contribute to an integrated, multiplex surveillance strategy in which hazardous areas are monitored for the presence of multiple arboviruses simultaneously.

## Methods

We considered Additional file 1: Table S1 from the systematic literature review by Esser et al. [22] to identify ecological risk factors associated with sustained circulation and spread of the six previously prioritized arboviruses (JEV, WNV, RVFV, CCHFV, TBEV and LIV). These factors included abiotic conditions (i.e. temperature, humidity and precipitation), vegetation cover, and the abundance of vectors and (reservoir) hosts such as migratory birds, livestock and deer. We then used nationwide continuous data on these ecological factors to construct hazard maps for the potential introduction and/or establishment of these arboviruses in the Netherlands. Factors that were not relevant for this country (e.g. elevation, presence of rice fields), or for which detailed information was not publicly available (e.g. point-to-point international livestock transport), were excluded from analysis. The available data allowed us to construct 9 hazard maps, 3 for the introduction of CCHFV, WNV, and JEV, and 6 for the establishment of each arbovirus (see below).

### Risk factors for introduction

Long-distance dispersal of infected vectors and/or wildlife is an important mechanism for the introduction and spread of arboviruses into novel areas [28]. We considered the relative abundance of bird species that migrate from Africa and/or the Mediterranean area to the Netherlands in spring as principal risk factor for the introduction of WNV and CCHFV (see Additional file 1: Table S1, for a list of included bird species). As these birds have overwintering and/or stopover sites in endemic regions, they may potentially be viraemic with WNV or carry *Hyalomma* ticks infected with CCHFV upon arrival in the Netherlands [29, 30]. Indeed, even though there are no established populations of *Hyalomma* ticks in the Netherlands, immature ticks are incidentally imported by migratory birds [31, 32]. Migratory birds may also spread JEV *via* overlapping migratory flyways with birds migrating from JEV-endemic regions in southeast Asia [33]. We therefore included the relative abundance of these bird species as risk factor for the introduction of JEV (see Additional file 1: Table S2).

Long-distance dispersal of infected livestock is arguably the most important mechanism for the introduction of RVFV and LIV into novel areas [28, 34]. In addition, livestock imported from CCHFV-endemic regions may also carry infected *Hyalomma* ticks [35]. However, data on point-to-point international transport of livestock is commercially and socially sensitive and was therefore not made available by competent bodies for this study, which precluded the construction of introduction risk maps for RVFV and LIV. Host movement also plays an important role in the spread of TBEV throughout Europe [36, 37], but as this virus emerged in the Netherlands while this study was ongoing, we limited our analysis to estimating the establishment of TBEV.

### Risk factors for establishment

For the mosquito-borne viruses, we included the following risk factors for establishment: (i) abundance of competent vectors; (ii) abundance of competent reservoir hosts; and (iii) suitable climatic conditions (Table 1). Competent mosquito species that have been prioritized as a veterinary and public health concern for the Netherlands, based on their local occurrence and vector status, include *Aedes vexans* for RVFV and *Culex pipiens* for WNV and RVFV [38–40]. More recently, European *Cx. pipiens* mosquitoes were also shown to be competent vectors for JEV [5]. We estimated the abundance of these two mosquito species across the Netherlands using random forest models following the methods described in Ibañez-Justicia & Cianci [41] and using the data reported in Ibañez-Justicia et al. [42]. Detailed information on these methods and the ecological variables used can be found in Additional file 2: Text S1.

**Table 1:** Ecological risk factors associated with the spread and sustained circulation of three mosquito-borne viruses (i.e. RVFV, JEV and WNV) and three tick-borne viruses (TBEV, LIV and CCHFV) that were included for the development of hazard maps for arbovirus introduction and establishment

	Introduction risk factors	Establishment risk factors		
		Abiotic	Host	Vector
Mosquito-borne viruses				
RVFV	na	Positive effect of TG from April to October	Abundance of ruminant livestock (i.e. sheep, goat, and cattle)	*Culex* and *Aedes* abundance
JEV	Abundance of birds with overlapping migratory flyways with conspecifics from JEV-endemic areas in Asia	Number of days with TX ≥ 25 °C	Abundance of ardeid bird species and domestic pigs	*Culex* abundance
WNV	Abundance of bird species that migrate from Africa and/or the Mediterranean area to the Netherlands in spring	Positive effect of TG from April to October	Abundance of wetland birds, crow, jackdaw, magpie, pigeon, and house sparrow	*Culex* abundance
Tick-borne viruses				
TBEV	na	Slope of TG decrease from August to October Slope of TG increase from March to May Positive effect of UG from April to October	Presence of deer and free-ranging livestock (i.e. cattle, sheep, goat, horse)	Suitable habitat for *I. ricinus* ticks
LIV	na	Slope of TG decrease from August to October Slope of TG increase from March to May Positive effect of UG from April to October	Abundance of sheep	Suitable habitat for *I. ricinus* ticks
CCHFV	Abundance of bird species that migrate from Africa and/or the Mediterranean area to the Netherlands in spring	Negative effect of RH Positive effect of TG from April to October	Abundance of livestock (i.e. cattle, horse, sheep, goat)	Suitable habitat for *H. marginatum* ticks

Choice of the below listed factors was based upon the systematic review of Esser et al. [22]

*Abbreviations*: UG, 24 h average relative humidity; RH, 24 h sum of precipitation; TG, 24 h average temperature; TX, 24 h maximum temperature

Different reservoir hosts are involved in the transmission cycle of each of the three mosquito-borne viruses. Domestic ruminants are the most important reservoir host for RVFV [34]. We therefore included the abundance of cattle, sheep, and goats as risk factor for RVFV establishment. Pigs and horses do not play a significant role in RVFV epidemiology [43, 44], whereas the role of small mammals and ruminant wildlife, such as deer, in virus maintenance remains unclear [45, 46]. These animals were therefore not included as risk factors for the establishment of RVFV. For JEV, Ardeid birds and pigs are the principal reservoir hosts [33]. Hence, we included the local abundance of Ardeid bird species that occur in the Netherlands (see Additional file 1: Table S3) and the abundance of pigs as risk factors for JEV establishment. West Nile virus is a multi-host pathogen that is maintained in a bird-mosquito transmission cycle [47]. Although the reservoir competence of many European bird species for WNV remains largely unknown, past outbreaks have often occurred near wetland areas, where large numbers of wetland birds and ornithophilic mosquitoes concentrate [48, 49]. In addition, experimental studies have shown that the European carrion crow (*Corvus corone*), the jackdaw (*Coloeus monedula*), the magpie (*Pica pica*), the rock pigeon (*Columba livia*) and the house sparrow (*Passer domesticus*) are all highly susceptible to WNV infection and are competent reservoir hosts [50–54]. We therefore included the relative abundance of wetland bird species and that of crow, jackdaw, magpie, rock pigeon and house sparrow as risk factors for WNV establishment (see Additional file 1: Table S4).

Temperature, humidity, and precipitation have all been associated with outbreaks of the three mosquito-borne viruses that we consider here [22]. However, we included only temperature as abiotic risk factor because of its direct impact on vector competence, biting rates, and the extrinsic incubation period of the virus [55, 56]. Other abiotic factors, such as precipitation or humidity, are indirectly related to virus circulation *via* their impact on mosquito abundance [57, 58], which we have modelled separately. Specifically, we included the average daily temperature during spring and summer (April to October) as risk factor for WNV and RVFV establishment, with higher temperatures corresponding with higher risk [56, 59]. For JEV, we included the number of days when the average daily temperature was at least 25 °C, a temperature limit above which JEV outbreaks occur [57].

For the tick-borne viruses, we included the following risk factors for establishment: (i) suitable tick habitat; (ii) presence of key host species for adult ticks and/or virus transmission; and (iii) abiotic conditions that facilitate virus transmission and/or tick development. Ticks are very sensitive to abiotic conditions and their survival is directly related to vegetation cover and leaf litter, which protects them from desiccation or freezing [60]. The principal vector of TBEV and LIV in Europe, *Ixodes ricinus*, occurs in a wide range of habitats, but it is typically found in woodlands and forests with thick undergrowth [61, 62]. Indeed, TBE incidence is positively correlated with the proportion of broad-leafed, mixed, and coniferous forest stands, which also provide habitat for small rodents that function as amplifying hosts for TBEV [63–66]. In contrast, the most prominent European vector of CCHFV, *Hyalomma marginatum*, prefers open country habitat [62], with clinical cases of CCHF being positively correlated with the proportion of shrub or grassland cover and with habitat fragmentation in agricultural areas [8, 26, 67]. We therefore included different land-use types for *I. ricinus* and *H. marginatum* in our analysis and scored these on a scale of 1 to 3, with higher values corresponding to more suitable habitat (see Additional file 1: Table S5).

Large herbivores are final hosts of adult *I. ricinus* and *H. marginatum* ticks, and are also directly involved as reservoir host in the transmission cycle of LIV and CCHFV [35, 36, 68]. In the Netherlands, deer presence rather than abundance best explains *I. ricinus* density [69]. In areas where deer and other wild herbivores are absent, free-ranging livestock that are used for nature management may instead maintain tick populations by feeding adult ticks [70]. We therefore used the presence of deer (i.e. roe deer *Capreolus capreolus*, fallow deer *Dama dama*, red deer *Cervus elaphus*) and free-ranging livestock (i.e. cattle, sheep, goat and horse) as a risk factor for high densities of *I. ricinus* and hence local circulation of TBEV. Louping ill virus most commonly occurs in upland habitats of the British Isles, where the red grouse (*Lagopus lagopus scotica*) is a competent transmission host, and the mountain hare (*Lepus timidus*) supports all three life stages of *I. ricinus* as well as non-viraemic transmission of LIV *via* co-feeding ticks [68]. Neither red grouse nor mountain hares are present in the Netherlands. Their absence, however, does not preclude local circulation of LIV in this country; sheep are highly competent reservoir hosts and are capable of maintaining an enzootic cycle with *I. ricinus* ticks, even in the absence of other key hosts such as deer [68]. We therefore included sheep abundance as a risk factor for the establishment of LIV. Livestock and hares are also principal host species for adult *H. marginatum* ticks and act as amplifying hosts for CCHFV [35]. Since the European hare (*Lepus europaeus*) is present throughout the Netherlands, but local abundance data were not available, we only included the abundance of livestock (i.e. cattle, goat, sheep and horse) as a risk factor for CCHFV establishment.

Rapid autumnal cooling followed by rapid spring warming are considered to be key climatic conditions for the transmission of TBEV and LIV as it enables synchronous activity of, and hence co-feeding transmission between, infected *I. ricinus* nymphs and uninfected larvae [71, 72]. We therefore included the rate with which temperatures decreased in autumn and increased in spring as a risk factor for establishment of TBEV and LIV. Autumnal cooling was calculated as the slope of the average daily temperature decrease from August 1st to October 31st. Spring warming was calculated as the slope of the average daily temperature increase from March 1st to May 31st. As moist conditions are a controlling factor for the survival of *I. ricinus*, we also included a positive relationship with relative humidity [73]. In contrast, *H. marginatum* ticks are adapted to the warm climatic conditions of northern Africa and southern Europe [74]. Various modelling studies showed a northward shift in climate suitability of this species with increasing temperatures and decreasing rainfall as predicted under future climate change scenarios [75–77]. We therefore included a negative relationship with rainfall and a positive relationship with temperature during summer months (April to October) as risk factors for CCHFV establishment.

### Raw source data

We used climatic data from the Royal Netherlands Meteorological Institute (KNMI). Daily meteorological data of 38 stations from 1 January 2010 until 31 December 2015 were used to interpolate (Spline function) daily maps with full coverage of the Netherlands. We followed the KNMI protocol for interpolating daily meteorological data [78]. We included the following factors: 24 h average temperature (TG; 0.1 °C); 24 h maximum temperature (TX; 0.1 °C), 24 h sum of precipitation (RH; 0.1 mm); and 24 h average relative humidity (UG; %). To identify suitable tick habitat, we used the LGN7 dataset [79] for land-use in the Netherlands, which differentiates between 39 land-use types (Additional file 1: Table S5). Livestock abundance data was obtained from the 2015 livestock survey database (Landbouwtelling 2015; poll date 1 April 2015) as provided by the Netherlands Enterprise Agency (RVO). Presence of free-ranging livestock in nature reserves was provided by Wageningen Environmental Research. Data on the presence of roe deer and hares was obtained from the Dutch National Database Flora and Fauna [80]. Data on the abundance of birds during the breeding season (spring) were obtained from the Bird Atlas of the Netherlands, based on nationwide fieldwork in 2013–2015 (www.vogelatlas.nl). The abundance of rare bird species was estimated per 5 × 5 km grid square on a semi-quantitative ordinal scale (classes: 1–3; 4–10; 11–25; 26–100; 101–500; 501–1000 breeding pairs). The abundance of common bird species was quantified by using geostatistical modelling, based on bird counts during standardized timed visits in eight systematically selected 1 × 1 km grid squares per 5 × 5 km square, and a set of environmental variables. For details of field work methods and modelling techniques, see Sovon Vogelonderzoek Nederland [81]. As the relative importance of each bird species for virus transmission remains unclear, we weighted each bird species equally in our analyses. For rare bird species, we took the geometric mean per abundance class, and then normalized the abundance of each species between 0 and 100. For common bird species, we directly normalized the abundance data by assigning 100 to cells where the species was most common and 0 where it was absent.

### Construction of hazard maps

We used ‘static risk mapping’ (*sensu* [82]) to characterize the spatial variation in the above-described ecological risk factors for arbovirus circulation. First, we generated a grid of 5 × 5 km covering the Netherlands in ESRI ArcGIS 10.5 (ESRI 2017). Cells that had their centroids > 1 km away from land were excluded from the analyses to prevent edge effects. Each ecological risk factor was represented by a single GIS-layer that covered the entire grid. Values for each layer were averaged per cell and then normalized (0–100) over the entire grid to allow adding or subtracting in further analyses (see below). All individual layers are provided in Additional file 3: Figures S1–S19.

The introduction maps for CCHFV, WNV and JEV consisted of one GIS-layer each, i.e. the relative abundance of migratory birds (Table 1). In contrast, the establishment maps of the six arboviruses were constructed by combining (overlaying) multiple GIS-layers. For this, we first classified each of these layers (risk factors) as belonging to either abiotic conditions, vector abundance, or host availability (Table 1). Because the relative importance of each risk factor varies between endemic regions [22], it remains unclear which factor(s) will contribute most to virus circulation in the Netherlands. All layers (risk factors) within each of the groups (abiotic, vector and host) were therefore weighted equally in the analysis by averaging all values per grid cell. The three groups were then overlaid and weighted equally again to prevent bias towards one particular group for arbovirus establishment.

For example, in the case of CCHFV, the host layer consisted of livestock abundance, the vector layer consisted of suitable habitat for *H. marginatum*, whereas the abiotic layer was a combination of temperature and precipitation (Table 1). As we included a positive effect of temperature but a negative effect of precipitation, their values could not simply be averaged. We therefore subtracted the precipitation values from 100 to obtain a scale where higher values correspond with lower precipitation (which is favourable for *H. marginatum*). Temperature and precipitation values were then averaged per grid cell and normalized. The three abiotic, host, and vector layers were then averaged and normalized again to construct the establishment map (see Additional file 4: Figures S20–S25, for a schematic representation of the procedure).

The hazard maps illustrate spatial differences in optimal environmental conditions for virus circulation and therefore portray relative hazard rather than actual hazard. This relative hazard is expressed on a scale between 0 (low hazard) and 100 (high hazard) and is visualised on the maps with colours ranging from black (low hazard area) to red (high hazard area). Because TBEV emerged in the Netherlands while this study was ongoing, we compared our establishment map with locations where TBEV was detected in ticks, wildlife, and humans, and where serologically positive roe deer were found [83]. These data were obtained from the website of the National Institute for Public Health and the Environment (RIVM), to which the Dutch Wildlife Health Centre, ErasmusMC, LabMicTa, MPH Services (GGD), Wageningen University and Research, and Artemis One Health contributed. In addition, we compared our WNV establishment map with locations where serologically positive birds were recently reported [84].

## Results

**Fig. 1:**
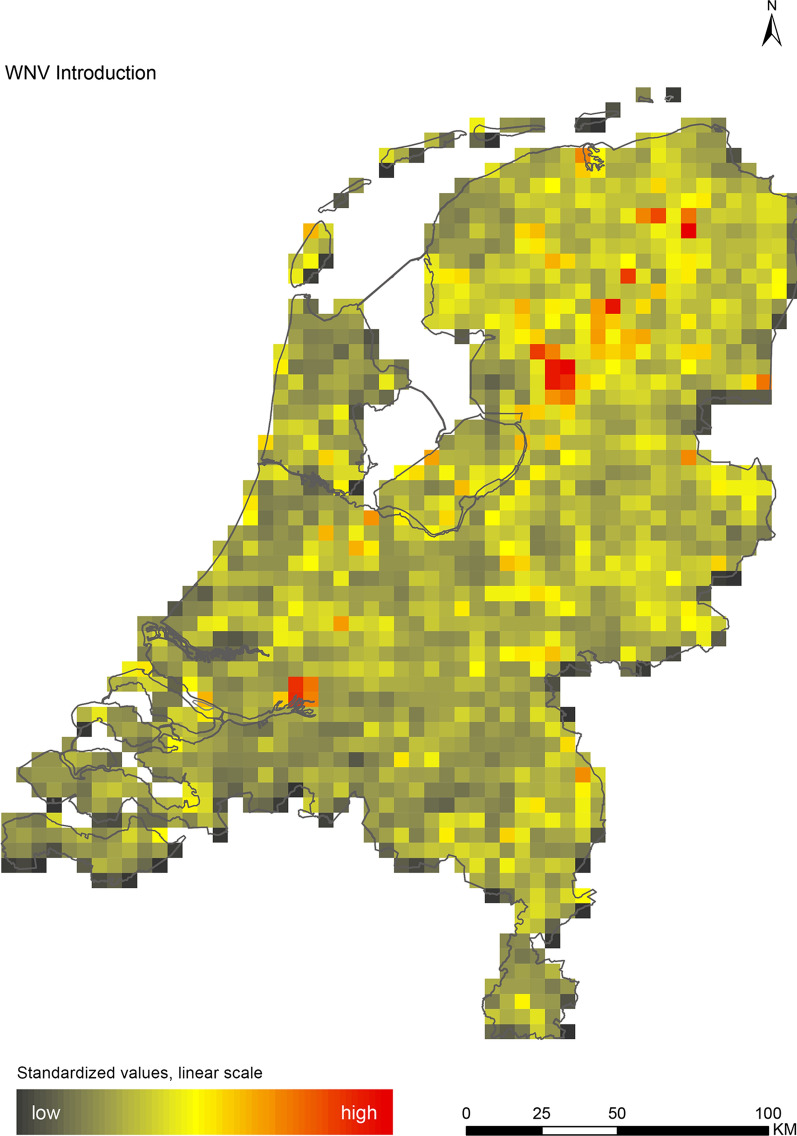
Hazard map for the introduction of West Nile virus (WNV) in the Netherlands

**Fig. 2:**
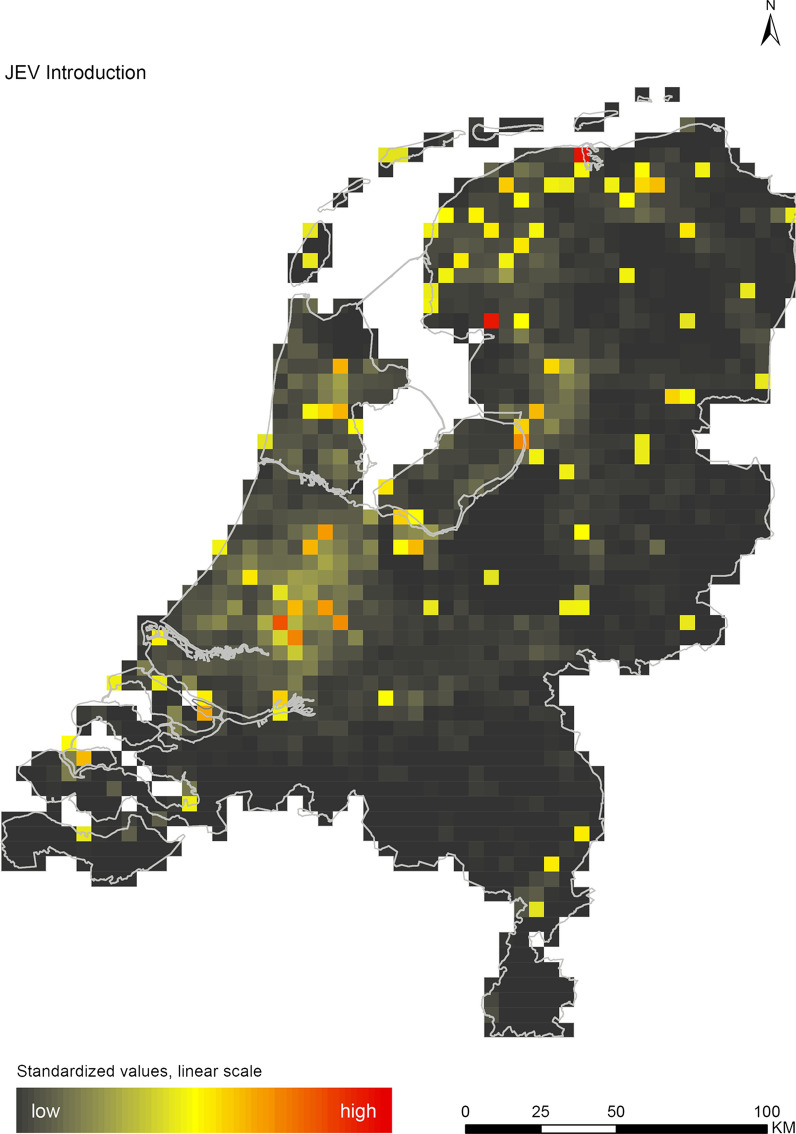
Hazard map for the introduction of Japanese encephalitis virus (JEV) in the Netherlands

**Fig. 3:**
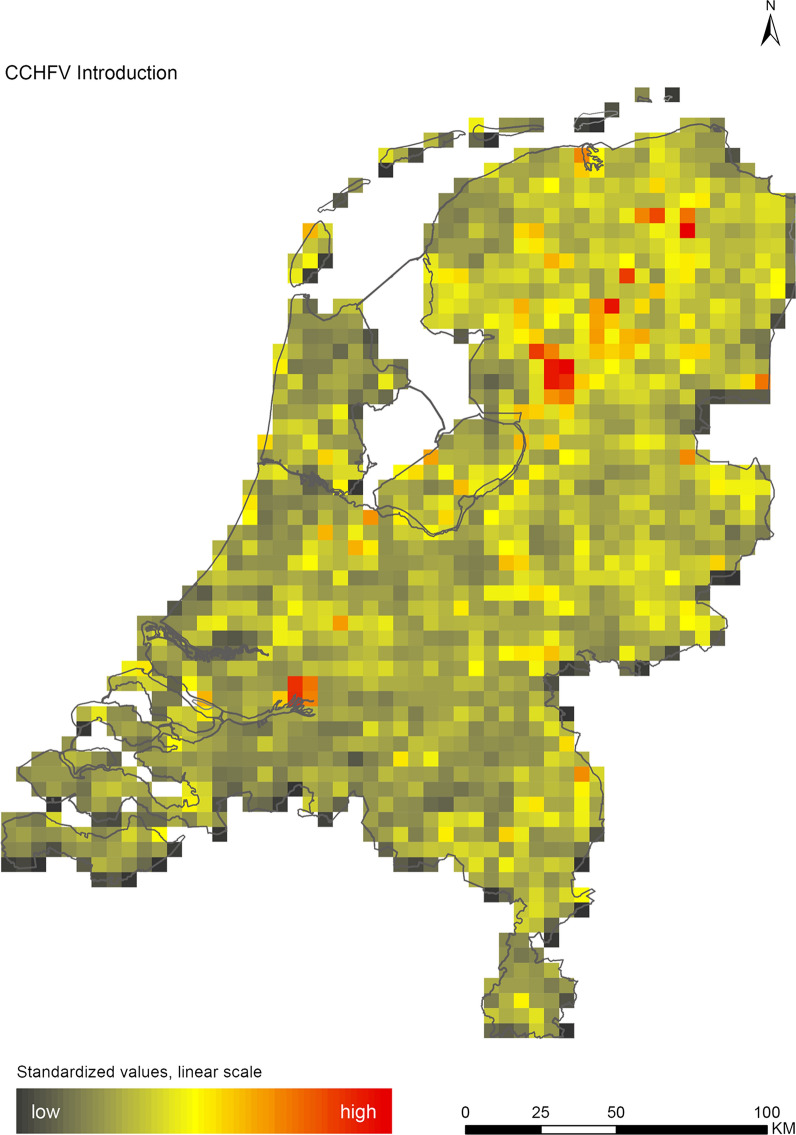
Hazard map for the introduction of Crimean-Congo haemorrhagic fever virus (CCHFV) in the Netherlands

**Fig. 4:**
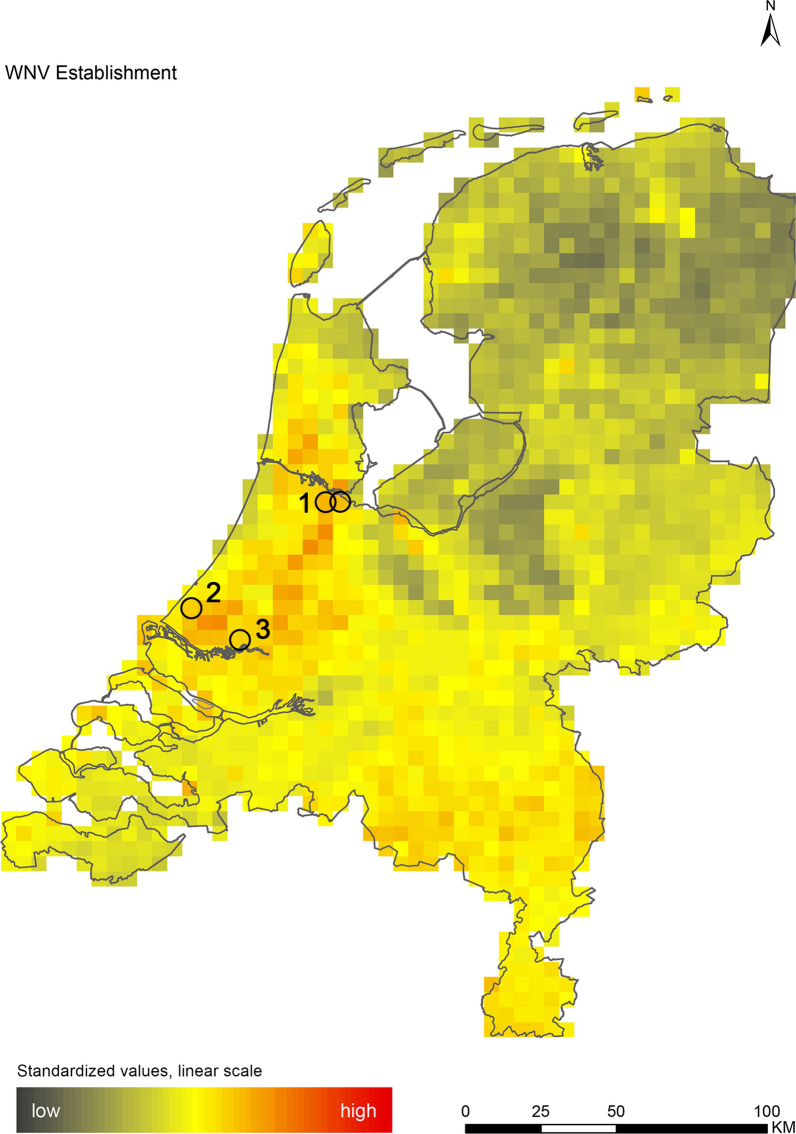
Hazard map for the establishment of West Nile virus (WNV) in the Netherlands. Locations where birds with WNV-neutralizing antibodies were caught [83] are indicated with black circles. Location 1: Amsterdam; Location 2: The Hague; Location 3: Rotterdam

**Fig. 5:**
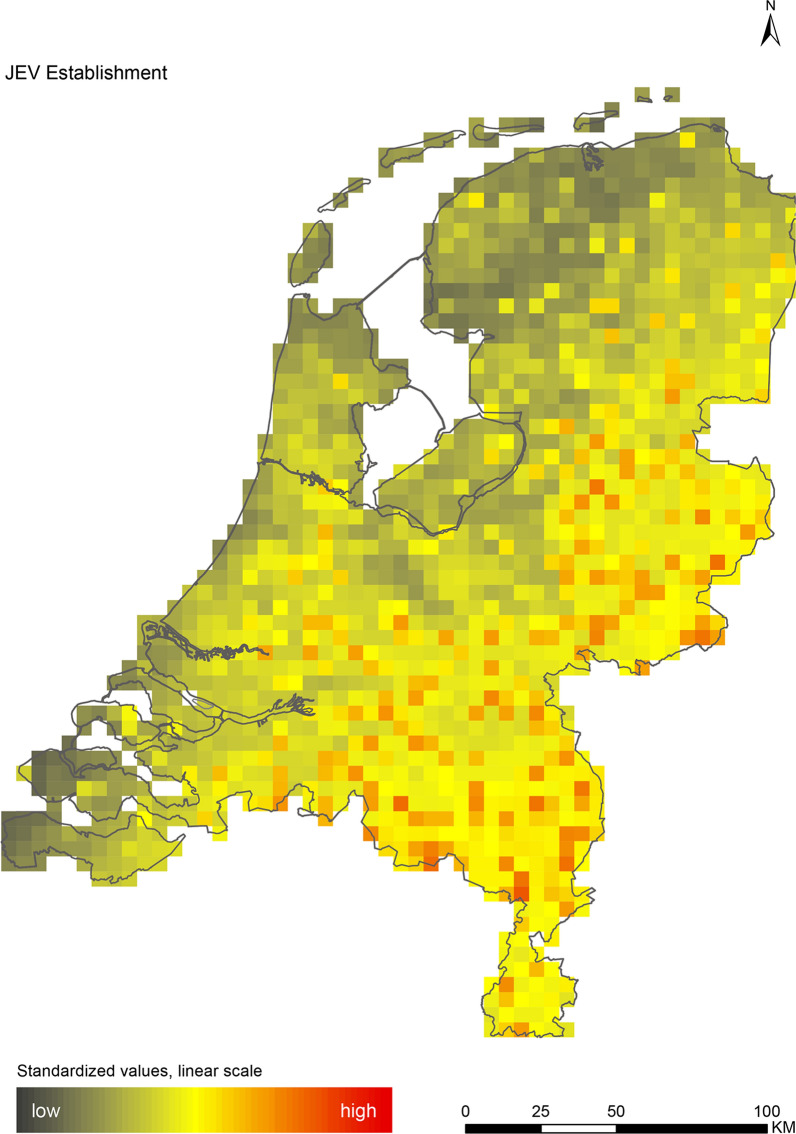
Hazard map for the establishment of Japanese encephalitis virus (JEV) in the Netherlands

**Fig. 6:**
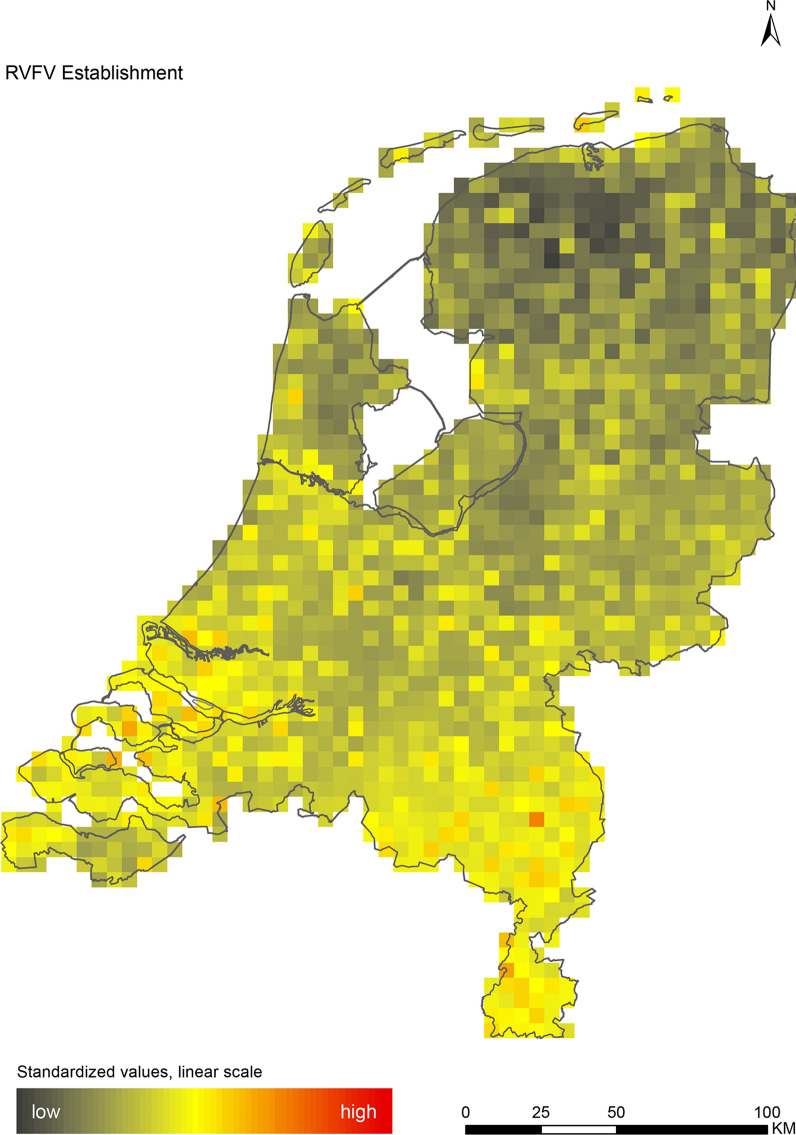
Hazard map for the establishment of Rift Valley fever virus (RVFV) in the Netherlands

**Fig. 7:**
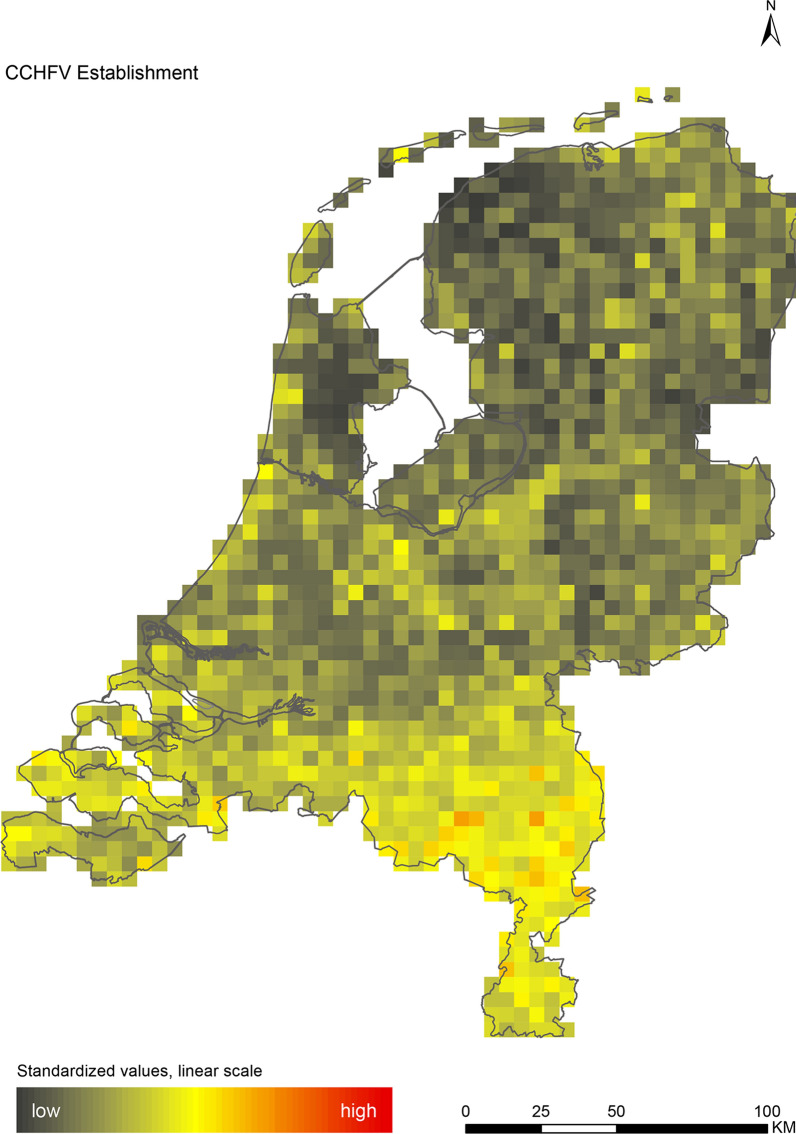
Hazard map for the establishment of Crimean-Congo haemorrhagic fever virus (CCHFV) in the Netherlands

**Fig. 8:**
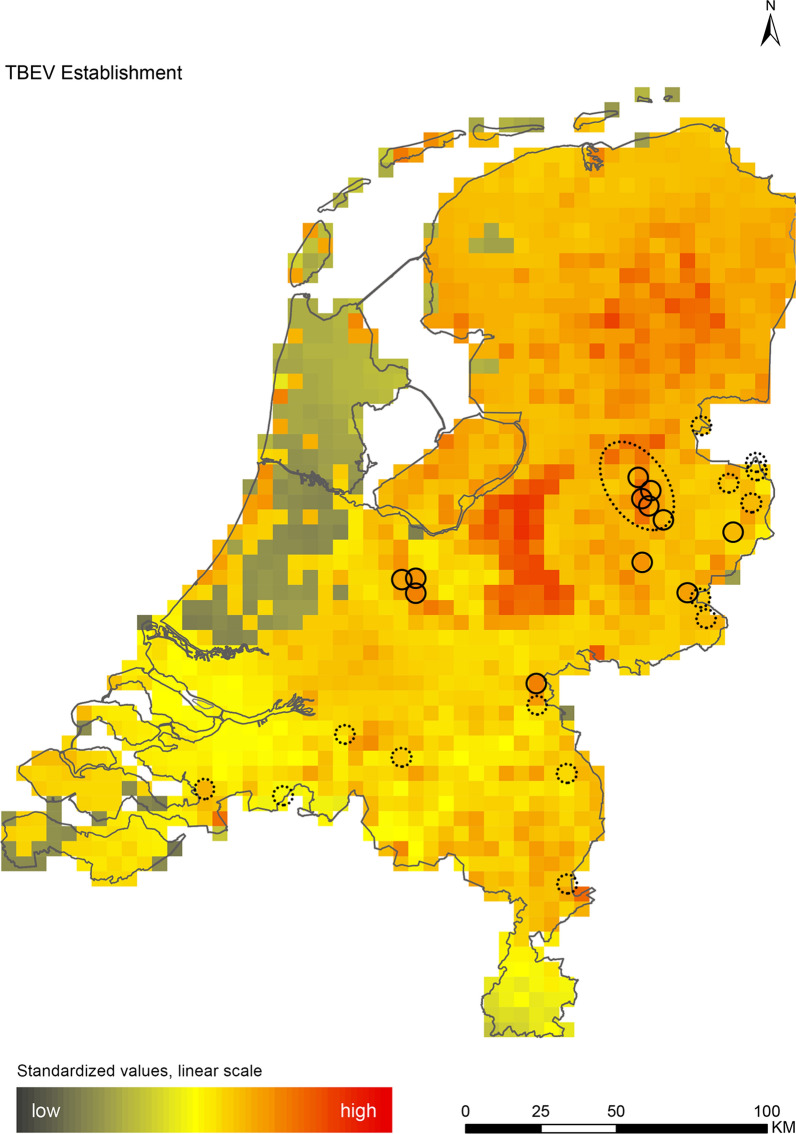
Hazard map for the establishment of tick-borne encephalitis virus (TBEV) in the Netherlands. Locations where TBEV-positive ticks, wildlife, and human cases were found are indicated with black circles. Locations where TBEV-seropositive wildlife were found are indicated with dashed circles [83]

**Fig. 9:**
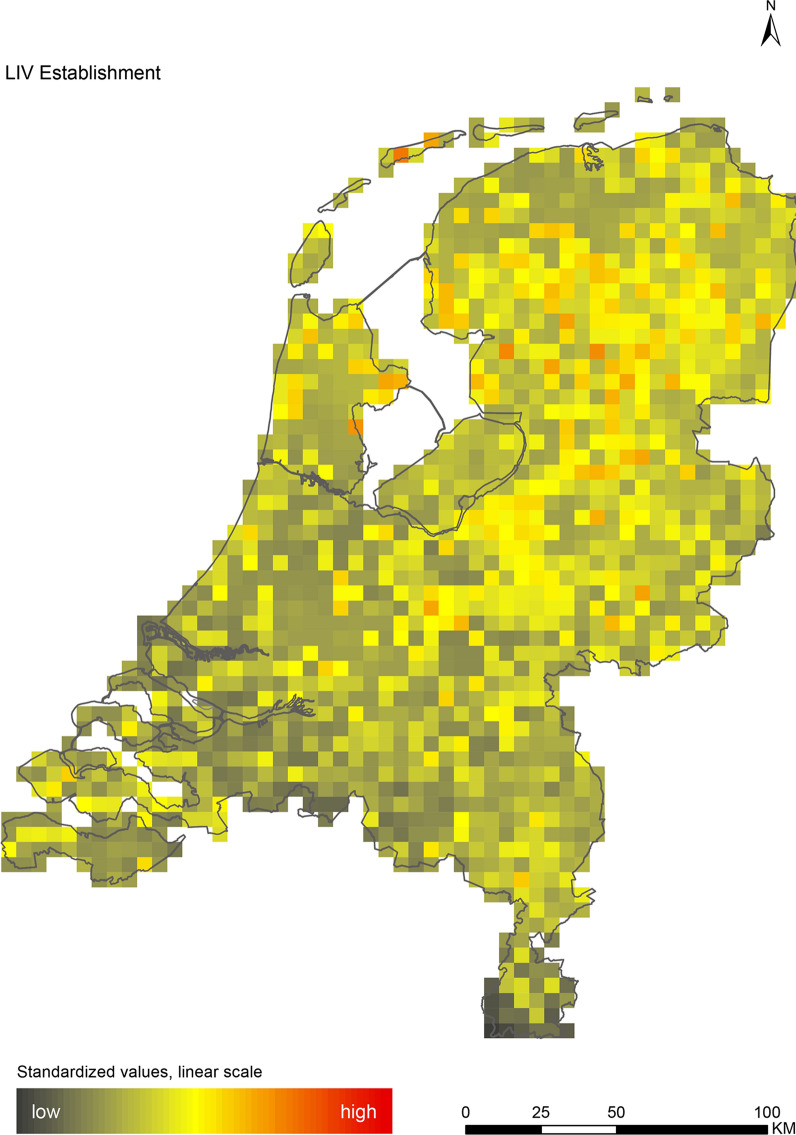
Hazard map for the establishment of louping-ill virus (LIV) in the Netherlands

**Fig. 10:**
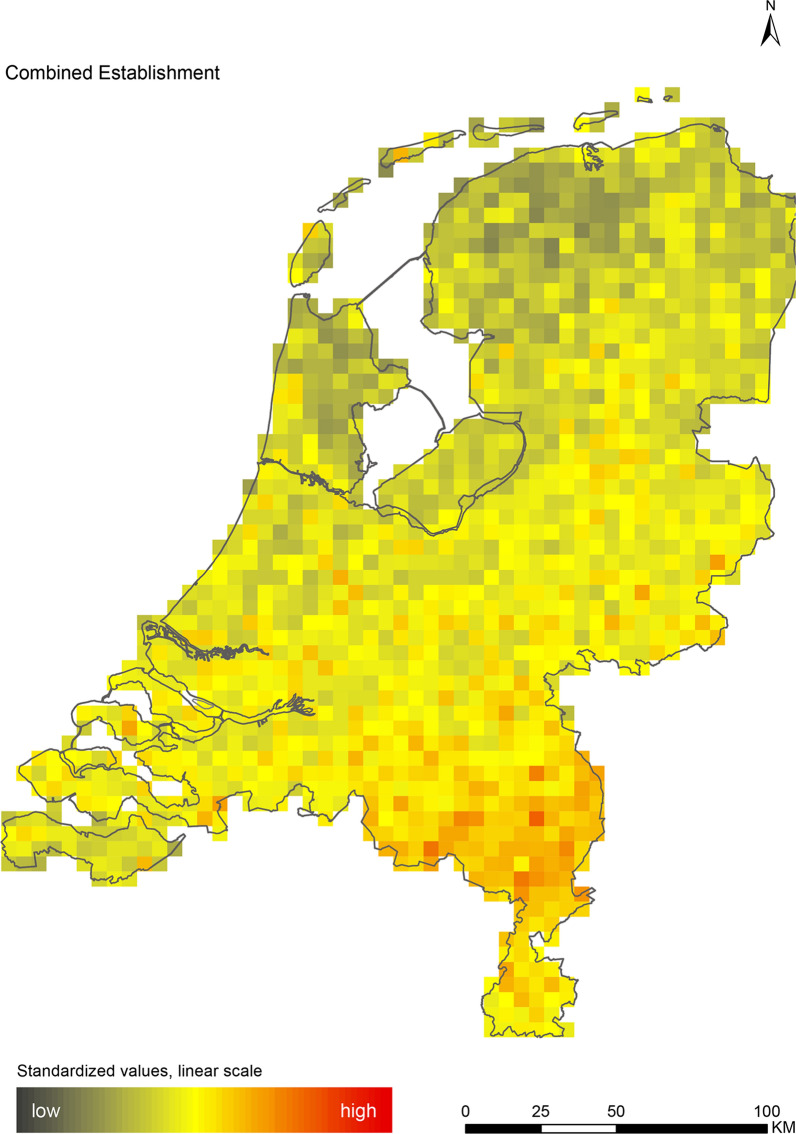
Combined establishment map for all six arboviruses (WNV, JEV, RVFV, TBEV, CCHFV and LIV) shows that the relative hazard is highest in the southern part of the Netherlands

The introduction maps highlight locations with large concentrations of migratory birds from appropriate source areas or species groups, which may either be infected with WNV or JEV, or carry CCHFV-infected *H. marginatum* ticks from endemic regions (Figs. 1, 2 and 3). The establishment maps indicated that for WNV, the southern and western part of the Netherlands are most suitable for endemic circulation (Fig. 4), while for JEV suitability was highest in the southern and eastern part of the country (Fig. 5). For RVFV and CCHFV, only few locations were classified as having a relatively high hazard for establishment, but they were all located in the south (Figs. 6, 7). Establishment hazard of TBEV was highest in nature areas in central, southern, and eastern parts of the country, where the combination of seasonal temperature profiles, suitable tick habitat and host availability are most likely to allow for co-feeding transmission between infected and uninfected ticks on rodent hosts (Fig. 8). In contrast, the hazard of LIV establishment was highest in the north of the country (Fig. 9). Overlaying all of the establishment maps showed that overall hazard of endemic arbovirus circulation was highest in the southern parts of the country; a region characterized by a warmer climate, which positively affects vectorial capacity and vector abundance [56, 75, 85] (Fig. 10).

As TBEV recently emerged in the Netherlands, we compared our establishment maps with the specific locations where the virus has been detected in ticks, humans, and wildlife and where seropositive roe deer were found [83]. Locations where PCR-positive ticks, wildlife and human cases have been found are all marked as high-hazard areas, while serological evidence is present in medium- to high-hazard areas, providing supportive evidence for the validity of our maps (Fig. 8). In addition, WNV-specific neutralizing antibodies were recently detected in birds (Eurasian coot *Fulica atra* and carrion crow) from Amsterdam, Rotterdam and The Hague [84]. While it is possible that these birds acquired WNV outside of the Netherlands, the cities in which they were found are located in the western part of the country, where the relative hazard for establishment was highest (Fig. 4).

## Discussion

We performed a spatial analysis of ecological risk factors for circulation of six arboviruses (WNV, JEV, RVFV, CCHFV, TBEV and LIV) to identify areas in the Netherlands with the highest potential for their introduction and subsequent establishment. We created introduction maps for WNV, JEV and CCHFV, and establishment maps for each of the six arboviruses (Figs. 1, 2, 3, 4, 5, 6, 7, 8 and 9). We stress that these maps portray spatial variation in relative hazard, i.e. arbovirus circulation is more likely in certain locations than in others, rather than actual hazard. That being said, the similarity between the predicted high-hazard areas and the locations where actual TBEV-cases and WNV-serologically positive birds were reported, provides some confidence that our spatial model, despite its relative simplicity, can be used to identify regions in the Netherlands where arbovirus emergence is most likely.

While TBEV appears to be locally established in the Netherlands, autochthonous WNV infections in mosquitoes or animals have yet to be reported, despite widespread availability of competent vectors [39] and expected introduction by viraemic birds [30, 86]. Temperature is regarded as the key limiting factor for WNV transmission in northern Europe [56, 87], but the virus is predicted to spread into this region *via* migratory birds under future climate change scenarios [88]. Similar predictions have been made for *H. marginatum*, which is both the main vector and reservoir host of CCHFV in Europe [75]. A population model showed that self-sustaining populations of this tick species were absent in areas where yearly accumulated temperatures drop below 3000–4000 °C [60]. In the Netherlands, the yearly accumulated temperature averaged 3751 °C during our study period (2010–2015), but is expected to rise above 4000 °C under the predicted temperature increase of 1 °C by 2030 [13]. However, this temperature limit was already exceeded in the exceptionally warm year of 2018, and the first imported *Hyalomma* ticks have already been found on migratory birds, horses and humans [31, 32]. Together, these findings warrant increased surveillance for TBEV, WNV and CCHFV.

In contrast, the potential introduction of RVFV, JEV and LIV is probably more dependent on human activities, such as trade and travel, rather than natural movement of hosts and/or vectors. For example, the red grouse plays an important role in the transmission cycle of LIV in the UK [89], but this species is absent in the Netherlands. Its closest relative, the black grouse *Lyrurus tetrix*, is critically endangered and has only a small population on the Sallandse Heuvelrug. Past introductions of LIV into other parts of Europe were likely due to international transport of infected sheep [90], and this is also the most plausible route of introduction for the Netherlands. Subsequent establishment of LIV is possible through a competent and abundant vector, *I. ricinus* [68]. Likewise, few bird species that arrive in the Netherlands in summer have overlapping migratory flyways with birds from JEV-endemic areas in Asia, resulting in limited potential for introduction when temperatures are suitable for viral replication. Such a long-distance migration might also reduce the viremia of these birds, so that they are no longer infectious upon arrival [91]. Alternatively, introduction of JEV as well as RVFV *via* infected mosquitoes that come with trade or air traffic is theoretically possible [91], but is considered to be less likely for the Netherlands than entry through (illegal) trade of birds (JEV) and livestock (RVFV) [92]. Access to animal trade data is of critical importance for mapping this hazard, and it is therefore extremely unfortunate that this information was not made available by the relevant bodies, who deemed it to be economically too sensitive.

The use of GIS-based models has become increasingly common in the field of spatial epidemiology to map the potential emergence of diseases in areas beyond their current distribution [82, 93–96]. Our approach is similar and generated useful results that are corroborated with recent findings of e.g. TBEV emergence. Further, overlap between some of the arboviruses’ high-hazard areas supports the implementation of integrated surveillance in regions where multiple arboviruses may emerge. On the other hand, it is difficult to weigh for differences in importance or effect sizes of different risk factors in our analyses at this stage. More experimental research is required to disentangle the effects of the different, often confounded environmental factors and to estimate their relative contribution to virus circulation. However, it is relatively easy to include a weighting factor in our analyses, or to add additional layers (e.g. risk for human exposure *via* recreation) so that the accuracy of the predictions can be improved. These predictions also need to be tested, and such a validation phase is a fundamental requirement to improve our understanding of the underlying causal mechanisms driving these spatial patterns. Testing the accuracy of these predictions will be a challenge, as most of the arboviruses considered here are still presumed absent from the Netherlands (i.e. CCHF, LIV, JEV, WNV and RVFV). However, the recent emergence of TBEV and the closely to WNV-related Usutu virus [15], might offer some prospect for testing these predictions. Indeed, the establishment pattern of USUV in the Netherlands shows some resemblance to the establishment map for WNV, with a gradual northwest-oriented spread from the southeast of the Netherlands [15].

## Conclusions

The use of spatial models has become a key method to map the environmental suitability for arbovirus circulation and to target surveillance in regions of potential emergence. Our analyses and the generated hazard maps show that there is spatial clustering of areas with either a relatively low or high potential for arbovirus introduction and/or establishment in the Netherlands. Importantly, some of these high-hazard areas overlap. Our combined map, showing the summed hazard for all six arboviruses per cell, shows that overall hazard is highest in the southern part of the country. Sampling of vectors and sentinel hosts should be focused in these key priority areas, where several arboviruses may emerge. Such targeted sampling increases the efficient use of limited resources for surveillance. Thus, the construction and subsequent overlaying of multiple hazard maps provides a promising approach for an integrated, cost-efficient, multiplex-surveillance strategy that targets multiple arboviruses simultaneously.

## Supplementary information

**Additional file 1: Table S1.** Bird species that migrate from Africa and/or the Mediterranean area to North-western Europe during spring. **Table S2.** Bird species that have overlapping migratory flyways with conspecifics migrating from JEV-endemic regions in southeast Asia. **Table S3.** Ardeid bird species of the Netherlands. **Table S4.** Wetland bird species of the Netherlands. **Table S5.** Land cover classes of the Netherlands (LGN7) that were included as habitat for *Ixodes ricinus* and *Hyalomma marginatum* ticks. Habitat suitability ranged from 0 (not suitable) to 3 (very suitable).

**Additional file 2: Text S1.** Description of the methods used for modelling mosquito abundance, including information on mosquito data collection, the environmental data used and the statistical methods applied.

**Additional file 3: Figures S1–S19.** Individual layers (ecological risk factors) that were used for constructing the hazard maps.

**Additional file 4: Figures S20–S25.** Schematic representation of the steps taken to construct the hazard maps for the establishment of each of the six arboviruses.

## Data Availability

The abiotic data that support the findings of this study are available from the Royal Netherlands Meteorological Institute (KNMI). However, restrictions apply to the availability of the host and vector data (e.g. abundance of livestock and birds, land-use dataset), which were used under license for the current study, and so are not publicly available. Data are however available from the authors upon reasonable request and with permission of the parties from which these data were obtained.

## Abbreviations

CCHFV: Crimean-Congo haemorrhagic fever virus
JEV: Japanese encephalitis virus
LIV: Louping-ill virus
RVFV: Rift Valley fever virus
TBEV: Tick-borne encephalitis virus
WNV: West Nile virus
KNMI: Royal Netherlands Meteorological Institute
RH: 24-h sum of precipitation
TG: 24-h average temperature
TX: 24-h maximum temperature
UG: 24-h average relative humidity
