# Supplementary material for: Spatial risk analysis for the introduction and circulation of six arboviruses in the Netherlands
Source: Parasit Vectors. 2020 Sep 10;13:464. doi: 10.1186/s13071-020-04339-0 (PMC7488554; doi:10.1186/s13071-020-04339-0)
Supplement: Supplementary file 2 — Additional file 2: Text S1. Description of the methods used for modelling mosquito abundance, including information on mosquito data collection, the environmental data used and the statistical methods applied. [file 13071_2020_4339_MOESM2_ESM.doc]

**Random Forest modelling for *Culex pipiens/torrentium***

**A. Ibanez-Justicia 20-02-2017**

Species distribution modelling combines the occurrence or the abundance of species with environmental data and estimates the similarity of the conditions at any site based to the conditions at the locations of known occurrence of a phenomenon. A common application of this method is to predict habitat suitability of species with environmental data as predictors.

Here we describe the mosquito data collection, the environmental data used and the statistical methods applied for modelling the occurrence and abundance of *Cx. pipiens/torrentium* in the Netherlands.

Mosquito Data

Data collected during the NVS-mosquitoes program carried out from April to October 2010-2013 by the Dutch Centre for Monitoring of Vectors were used. The mosquitoes were captured using CO2 baited Mosquito Magnet Liberty Plus MM3100 (Woodstream® Co., Lititz, USA) [14]. The traps were randomly distributed in the country following the study design described in Ibañez-Justicia et al. (2014), with the following constraint: 40% of the traps were placed in urban areas, 40% in rural-agricultural areas and 20% in natural areas. Urban areas represent around 20% of the country total area; rural-agricultural areas and natural areas represent 60% and 20% of the country total area respectively. Urban areas were sampled to a greater extent because of their higher involvement in public health risks caused by mosquito vectors. One trap was placed at each location during one week, and each location was only sampled once. The data consisted of values of mosquito abundance collected in 778 geo-referenced point locations. For this study the data were also reclassified as presence and absence for each location.

Due to the difficulty of morphologically identifying *Cx. pipiens* and *Cx. torrentium* adult mosquitoes, specimens of this group were identified as *Cx. pipiens/torrentium*, but because of the recent outbreaks of WNV in Southern Europe (Balenghien et al., 2008) and the implication of members of the *Cx. pipiens* complex mosquitoes (*Cx. pipiens pipiens*, *Cx. pipiens molestus* or hybrids), and recent outbreaks of Sindbis virus and the implication of *Cx. torrentium* (Hesson et al., 2011), molecular investigations based on a real-time PCR version of Rudolf et al. (2013) are in progress with the goal of analyzing the majority of the *Cx. pipiens/torrentium* samples included in this study in order to provide information about the geographical distribution of the *Cx. pipiens* complex and *Cx. torrentium* in the Netherlands.

Since the spatial resolution used in the study is 1 km2, when a presence and an absence point were in the same square kilometre only the presence point was selected because presences inform about the places that are environmental suitable for a species, but absences do not necessary indicate the opposite [15]. This reduced the number of locations used in the analysis from 778 to 766.

Environmental variables

The environmental factors included in the analysis are the raster files commonly used for mosquito distribution modelling [16]. They were obtained from the MODIS sensor on NASA’s Terra and Aqua satellites [17] for 2000-2012. They were transformed applying the temporal Fourier analysis [18, 19] to summarise the images and to produce sets of data that capture important characteristics of the annual seasonality: the mean, the annual bi-annual and tri-annual amplitudes and phases, the maxima, minima and variances of the middle infra-red, day and night-time land surface temperature, the enhanced vegetation index and the normalized difference vegetation index signals [20]. Other environmental data used in this study are precipitation (WorldClim and CMORPH, 1950-2000), population density in year 2000 (compiled from the Gridded Population of the World Dataset), the digital elevation model (MODIS, 2012) and the Corine land cover map of 2006. A list of the Fourier components is provided in Table 1 and the environmental data are listed in Table 2. Predictor variables are organized as raster type ﬁles (TIF files, resolution 1 km2) and for each trap location the pixel values of the environmental variables were extracted.

Statistical analysis

Random (classification) forest modelling technique presence/absence data was applied: The accuracy was assessed using (i) sensitivity, i.e. the ability of a model to correctly identify known positive sites; (ii) specificity, i.e. the ability of a model to correctly identify known negative sites; (iii) the area under the curve, (AUC) that can be roughly interpreted as the probability that a model will correctly distinguish a true presence and a true absence [23].

The output produced was an environmental suitability indicator, expressed as a value between 0 (low suitability) and 1 (high suitability). The predictions are visualised in a map with colours ranging from red (high suitability) to blue (low suitability). A list of the most important variables used in the model is given based on the mean decrease in Gini index [21, 24].

Random (regression) forest was used to model the abundance of the species. The abundance data were transformed according to the formula log10(abundance+1) [25]. The predicted probability of occurrence, interpreted as probability of habitat suitability, is included in the predictors list for modelling the abundance of the species [26–30]. Accuracy was assessed through the mean squared error (MSE) and the R2, indicator of the goodness of fit of the model. The importance of the predictors was assessed using the Increase in Node Purity (INP). The analysis was performed with the software Vecmap demo version [31].

| RANDOM FOREST (CLASSIFICATION) PRESENCE/ABSENCE | |
| --- | --- |
| Input: Presence and Absence *Cx. pipiens/torrentium* between week 23 and week 42 (June until October) | |
| Abs 187  Pres 187  Total 374 | |
| 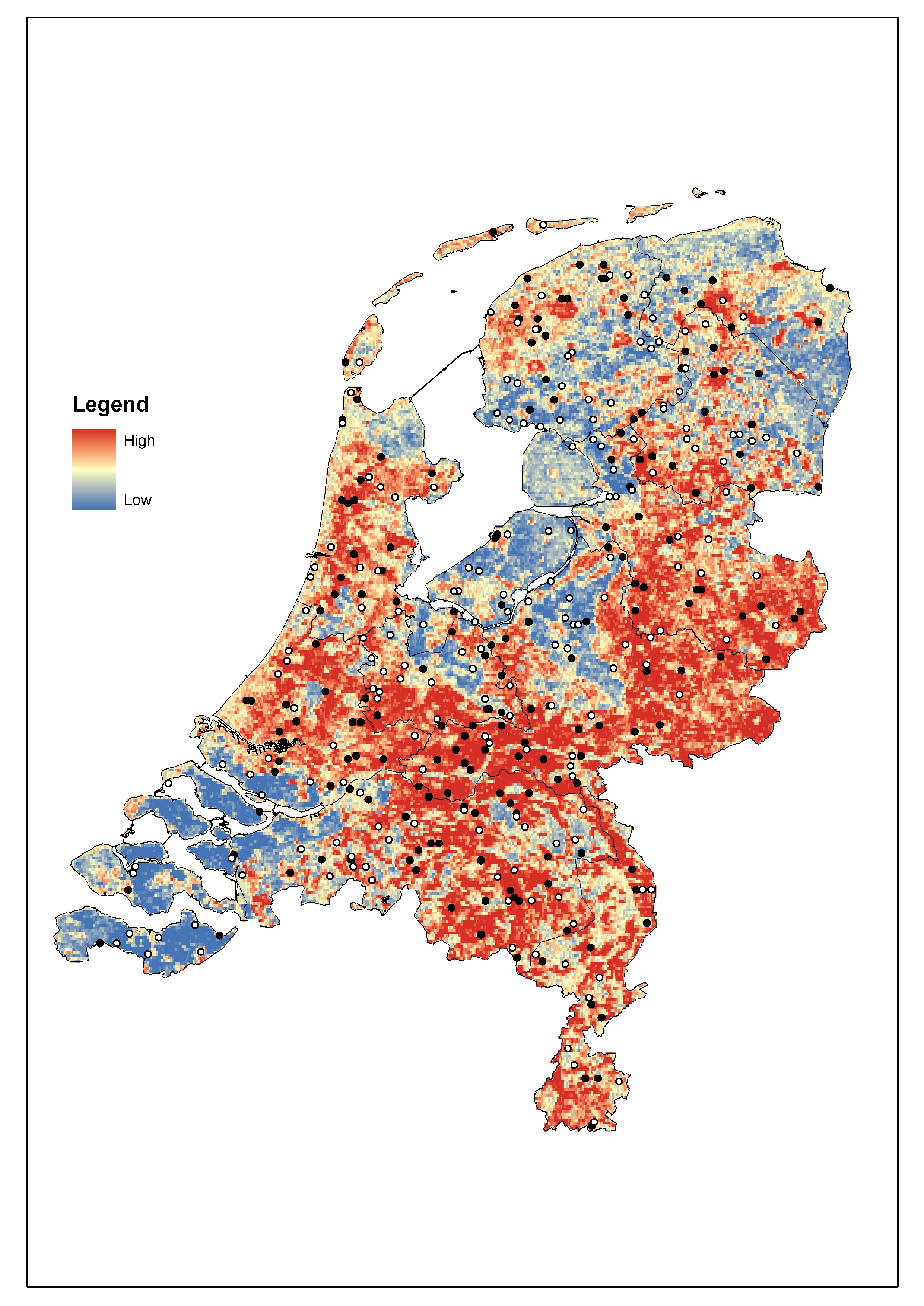 | |
| Cohen's Kappa:  AUC:  Sensitivity:  Specificity: | 0.1229  0.5718  0.5935  0.5657 |
| 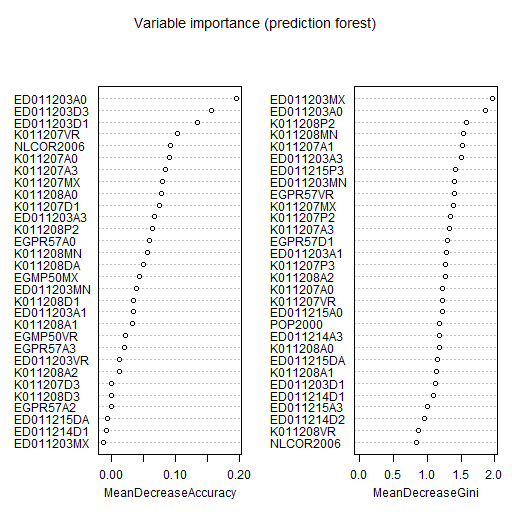 | |

| RANDOM FOREST (REGRESSION) ABUNDANCE | | | |
| --- | --- | --- | --- |
| Input: LOG10(N+1) abundance *Cx. pipiens/torrentium* between week 23 and week 42 (June until October) | | | |
| Abs 187  Pres 187  Total 374 | | | |
| 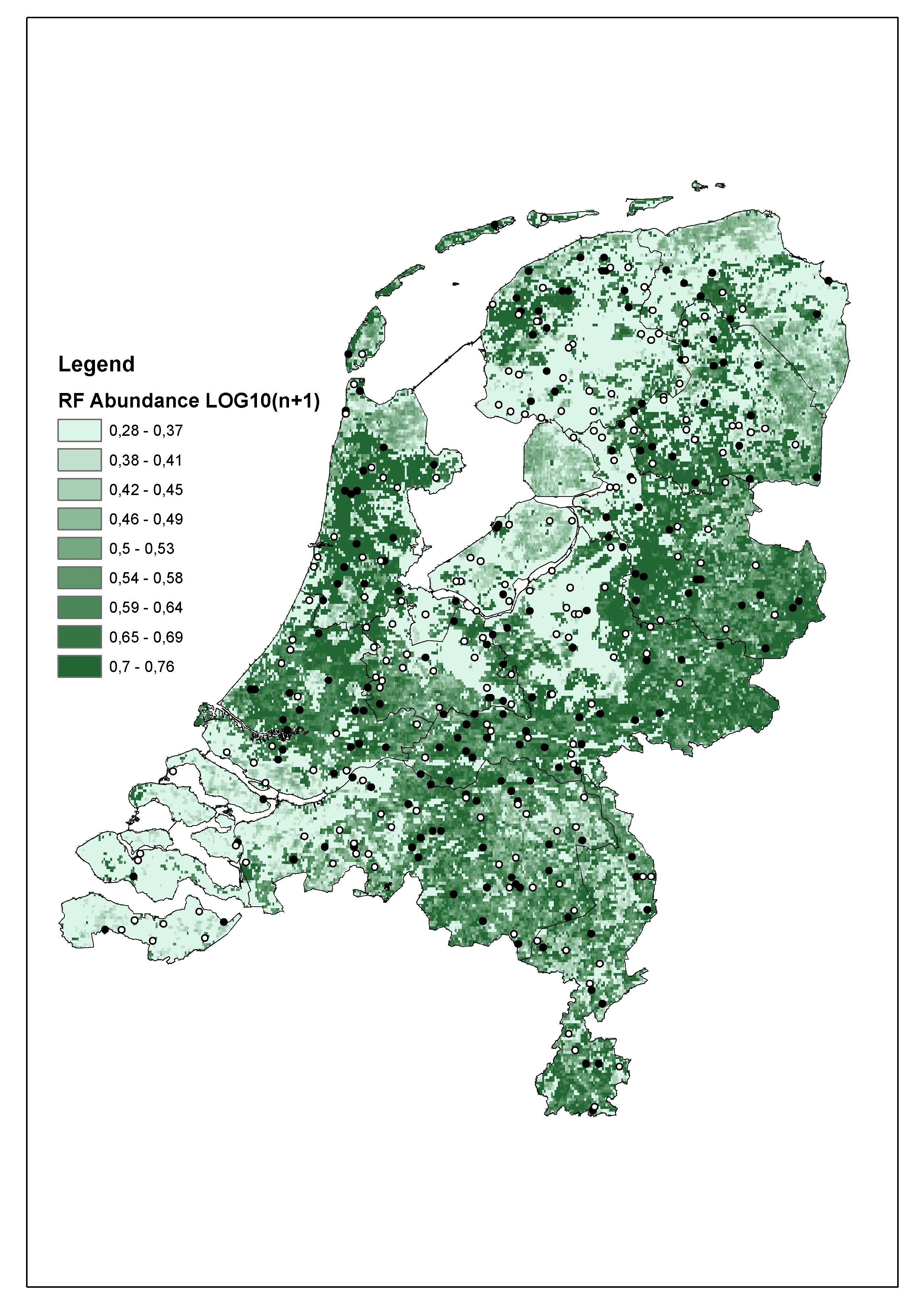 | | 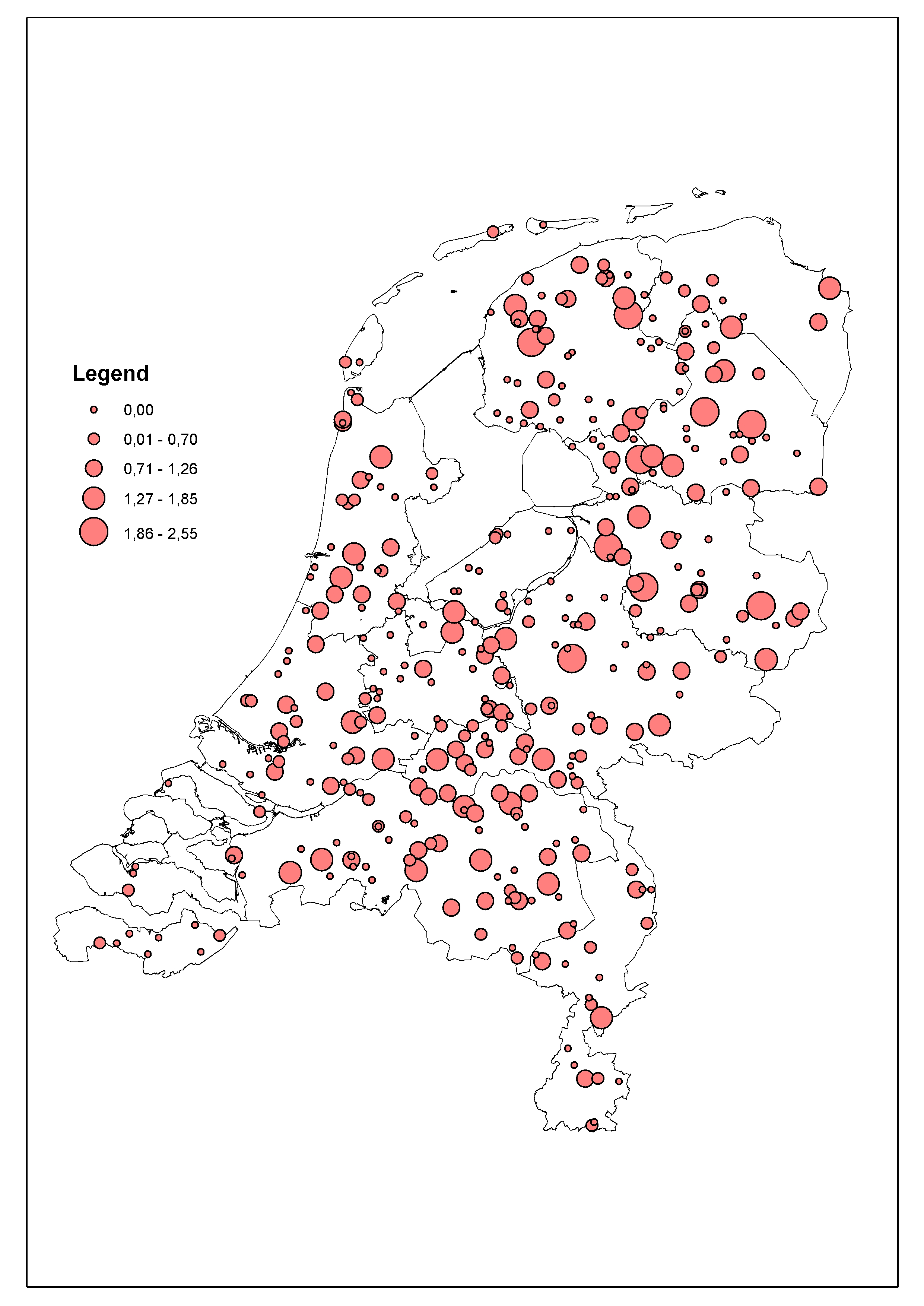 | |
| MSE  R^2^ | 0.2701  0.1405 | | |
| 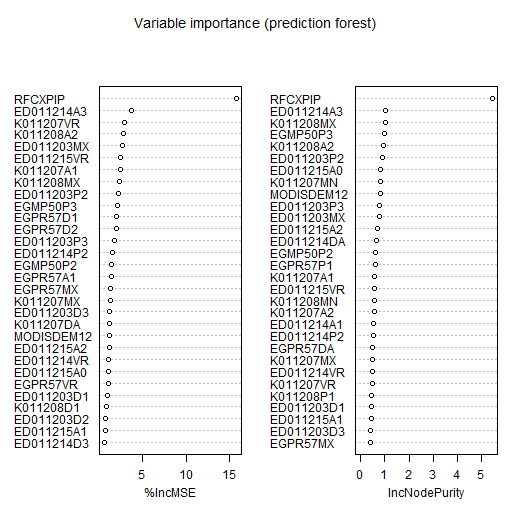 | | | 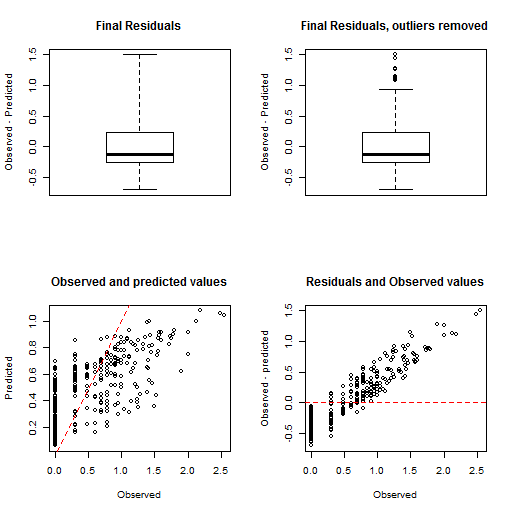 |

The mean squared error is high (MSE) and the coefficient of determination (R^2^) is low. By far, the most important variable is the estimate of the habitat suitability. Relationship between the observed abundance and the predicted abundance of *An. plumebus*, both *log_10_(n+1)* with *n*=number of mosquitoes.

Table 1 Fourier components from temporal Fourier analysis of an imagery time series. Component is the name used in Vecmap.

| Component | Description |
| --- | --- |
| A0 | Fourier mean for entire time series |
| MN | Minimum value |
| MX | Maximum value |
| A1 | Amplitude of annual cycle |
| A2 | Amplitude of bi-annual cycle |
| A3 | Amplitude of tri-annual cycle |
| VR | Total variance |
| P1 | Phase of annual cycle |
| P2 | Phase of bi-annual cycle |
| P3 | Phase of tri-annual cycle |
| D1 | Proportion of total variance due to annual cycle |
| D2 | Proportion of total variance due to bi-annual cycle |
| D3 | Proportion of total variance due to tri-annual and cycle |
| DA | Proportion of total variance due to all three cycles |

Table 2 Environmental predictor variables.

| Variables |
| --- |
| MODIS Middle Infra-red (MIR) |
| MODIS Day-time land surface temperature (DLST) |
| MODIS Night-time land surface temperature (NLST) |
| MODIS Enhanced vegetation index (EVI) |
| MODIS Normalised difference vegetation index(NDVI) |
| CMORPH precipitation |
| Worldclim precipitation |
| MODIS Digital elevation model (DEM) |
| Human population density |
| Corine land cover |

**Table 3. List of the most important variables on classification model** based on the mean decrease Gini index

| Variables |
| --- |
| MODIS Middle Infra-red (MIR) MX |
| MODIS Middle Infra-red (MIR) A0 |
| MODIS Night-time land surface temperature (NLST) P2 |
| MODIS Night-time land surface temperature (NLST) MN |
| MODIS Day-time land surface temperature (DLST) A1 |
| MODIS Middle Infra-red (MIR) A3 |
| MODIS Enhanced vegetation index (EVI) P3 |
| MODIS Middle Infra-red (MIR) MN |
| WORLDCLIM precipitation VR |
| MODIS Day-time land surface temperature (DLST) MX |
