# Supplementary material for: Spatial risk analysis for the introduction and circulation of six arboviruses in the Netherlands
Source: Parasit Vectors. 2020 Sep 10;13:464. doi: 10.1186/s13071-020-04339-0 (PMC7488554; doi:10.1186/s13071-020-04339-0)
Supplement: Supplementary file 3 — Additional file 3: Figures S1–S19. Individual layers (ecological risk factors) that were used for constructing the hazard maps. [file 13071_2020_4339_MOESM3_ESM.pdf]

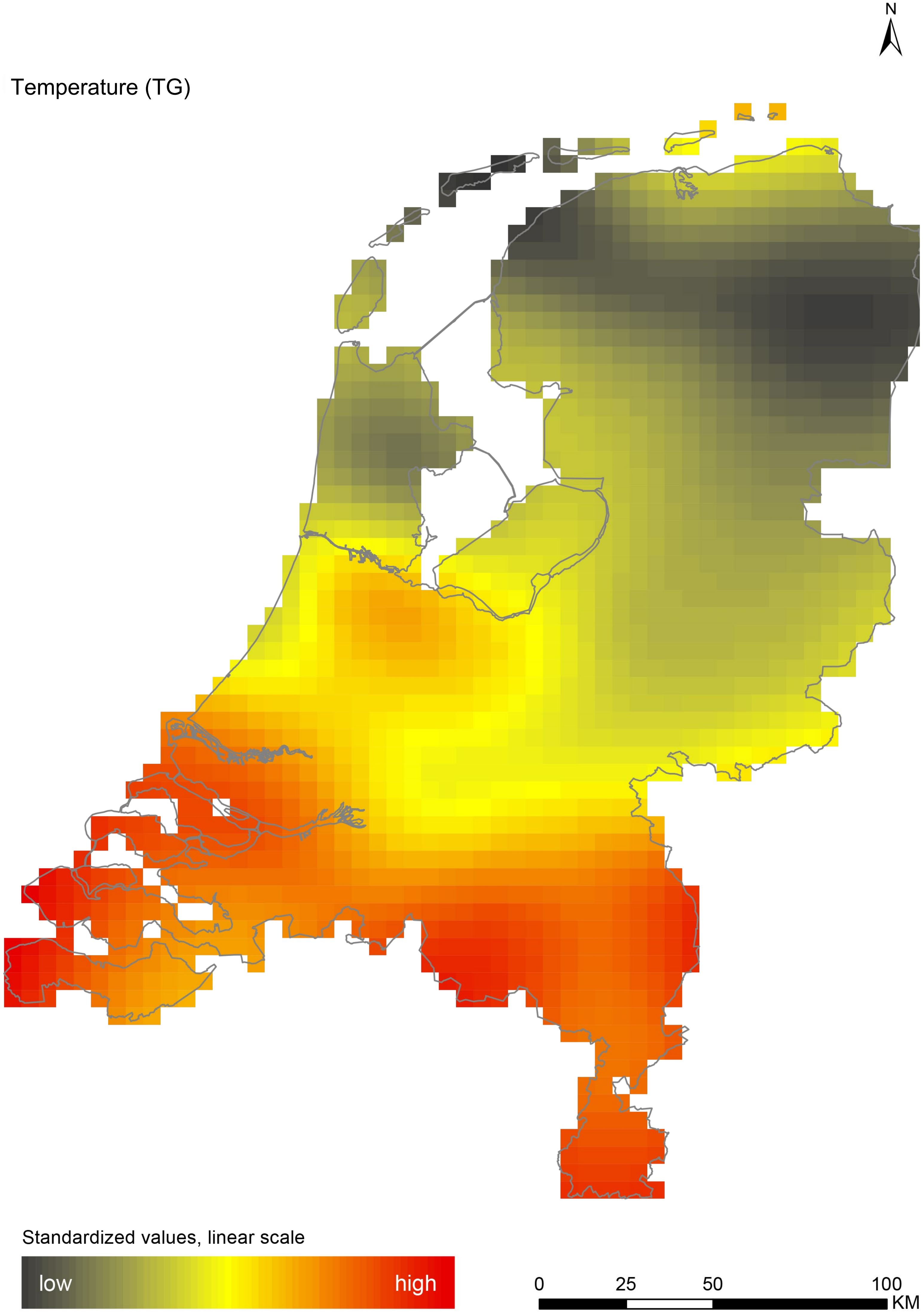
N
Temperature (TG)
Standardized values, linear scale
low
high
0
25
50
100
KM

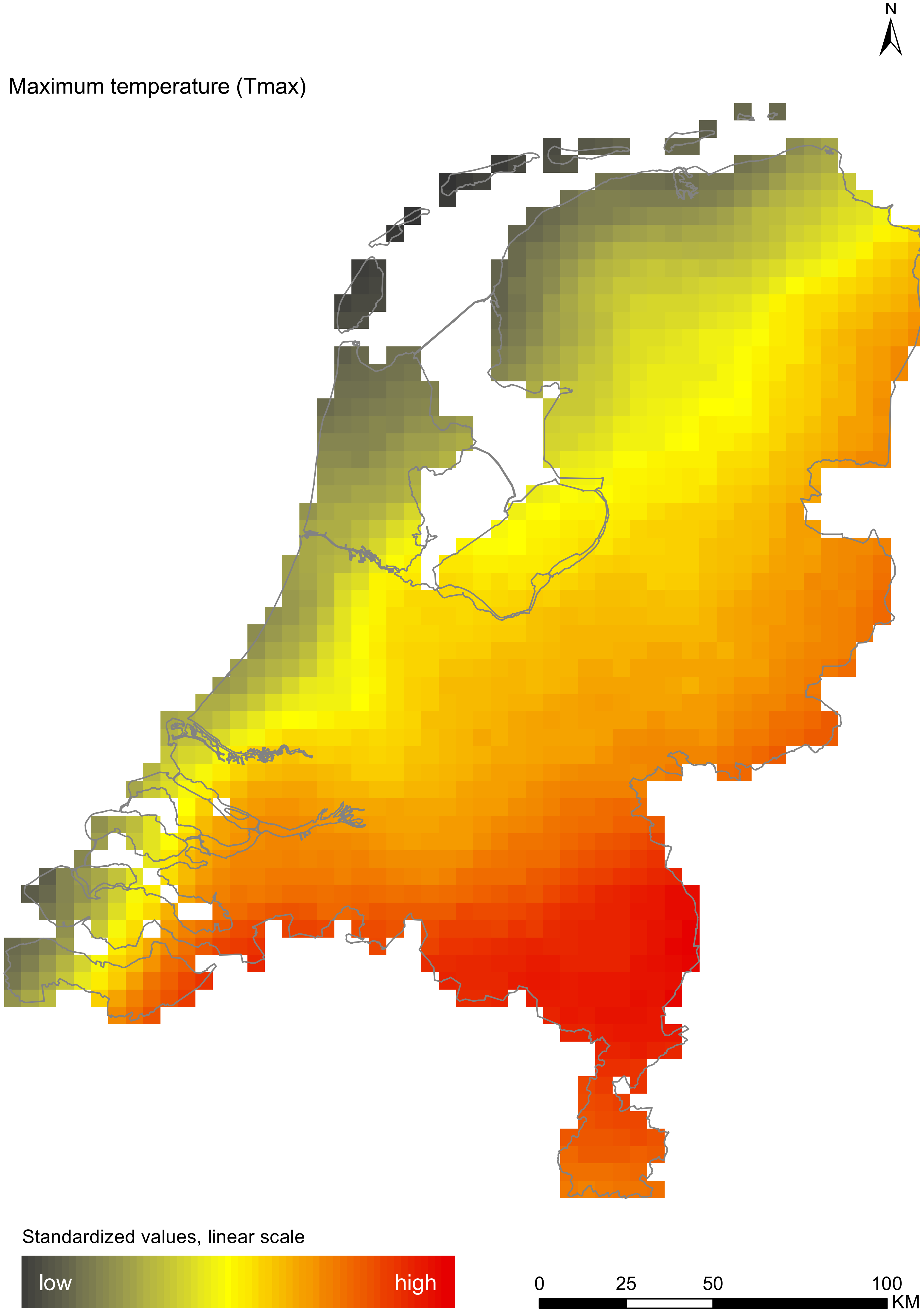

N
Maximum temperature (Tmax)
Standardized values, linear scale
low
high
0
25
50
100
KM

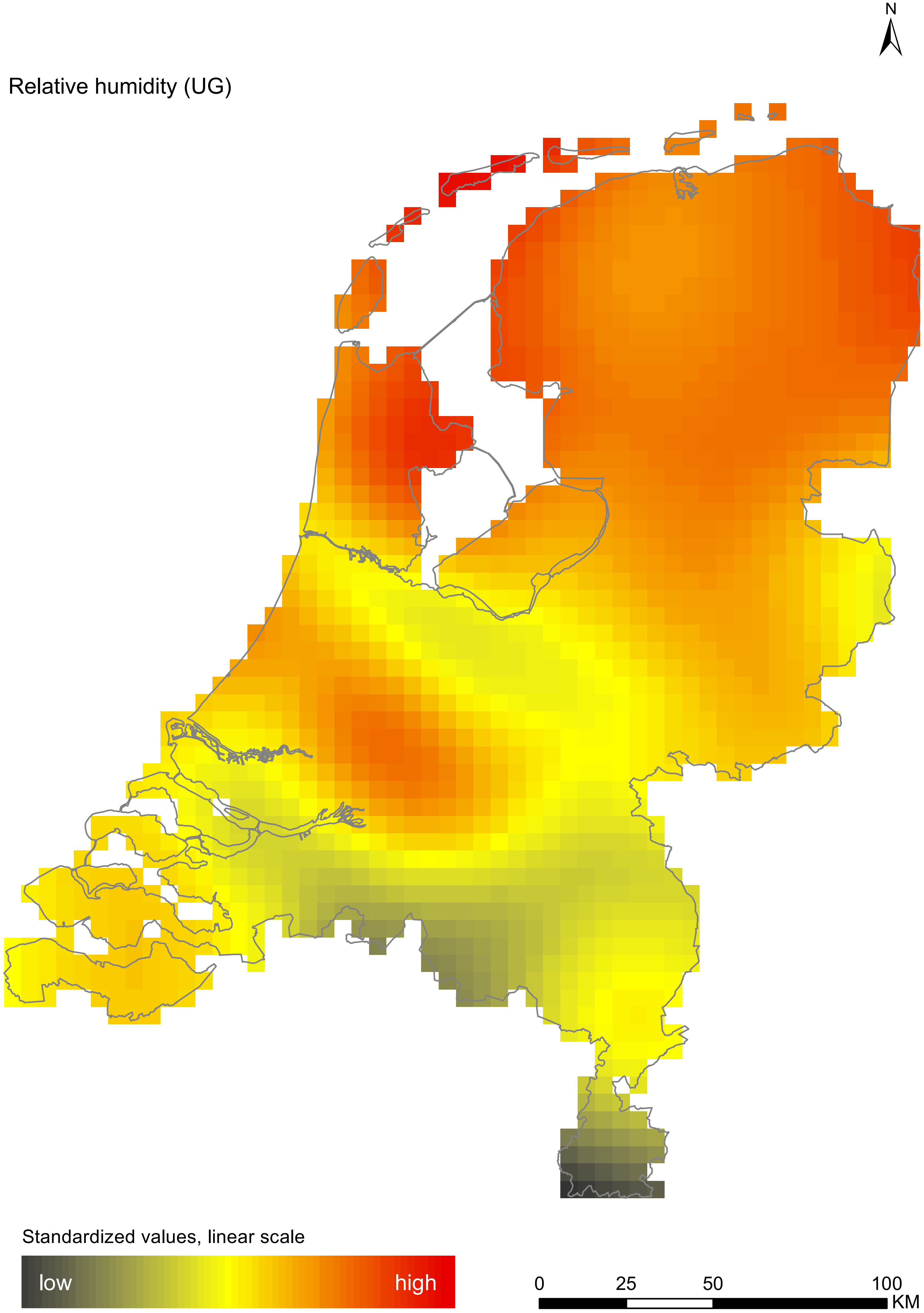

N
Relative humidity (UG)
Standardized values, linear scale
low
high
0
25
50
100
KM

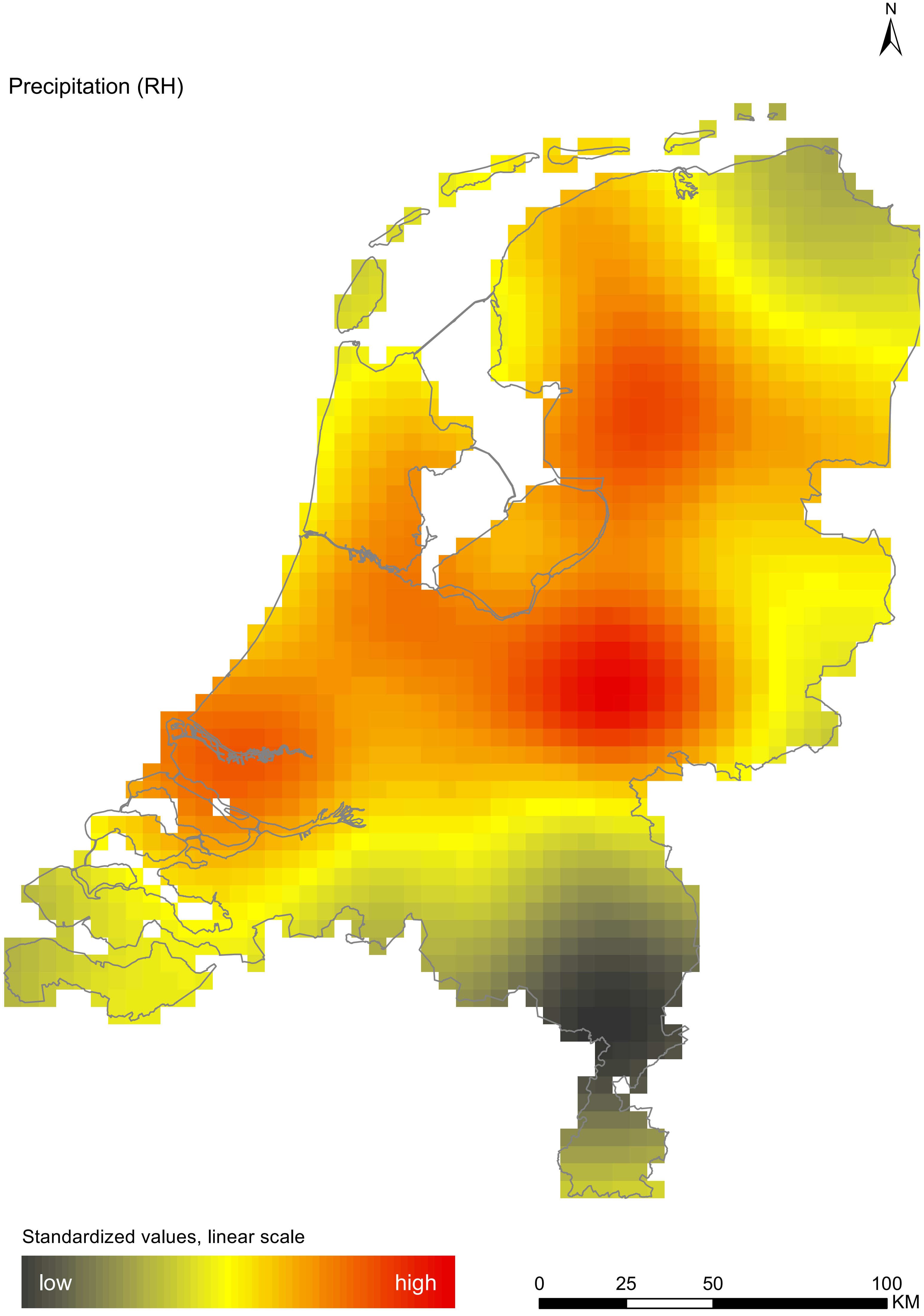
N
Precipitation (RH)
Standardized values, linear scale
low
high
0
25
50
100
KM

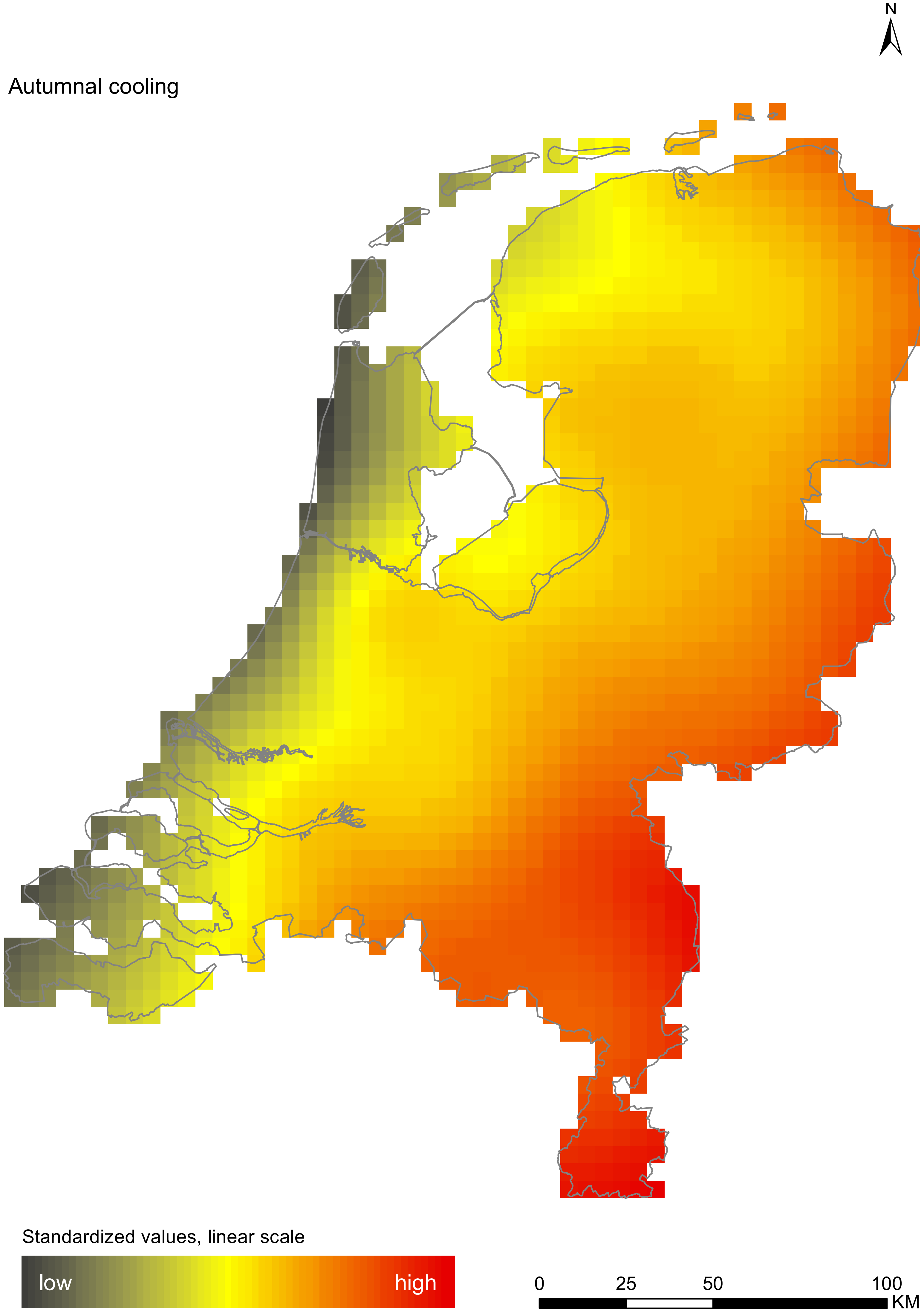
N
Autumnal cooling
Standardized values, linear scale
low
high
0
25
50
100
KM

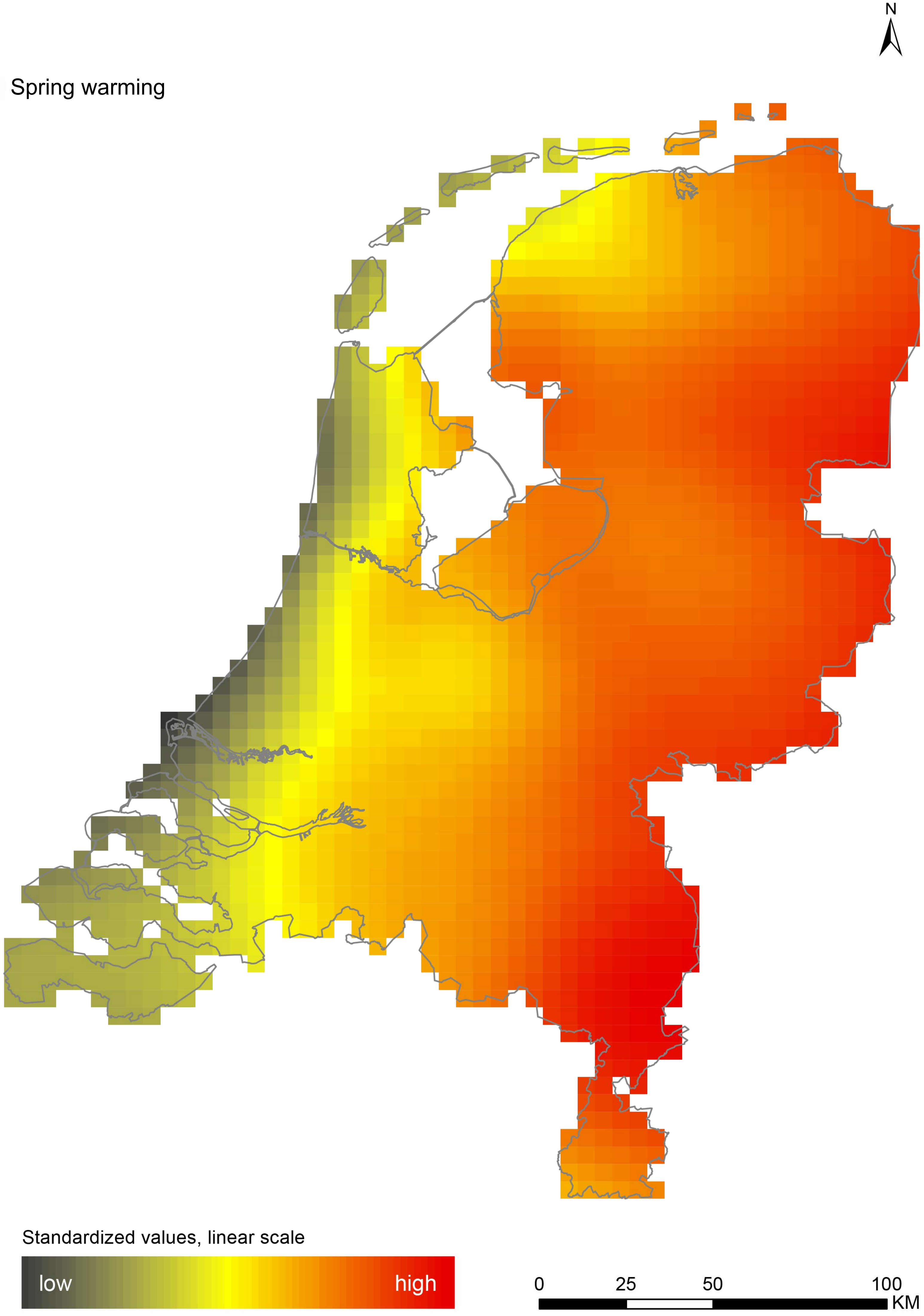
N
Spring warming
Standardized values, linear scale
low
high
0
25
50
100
KM

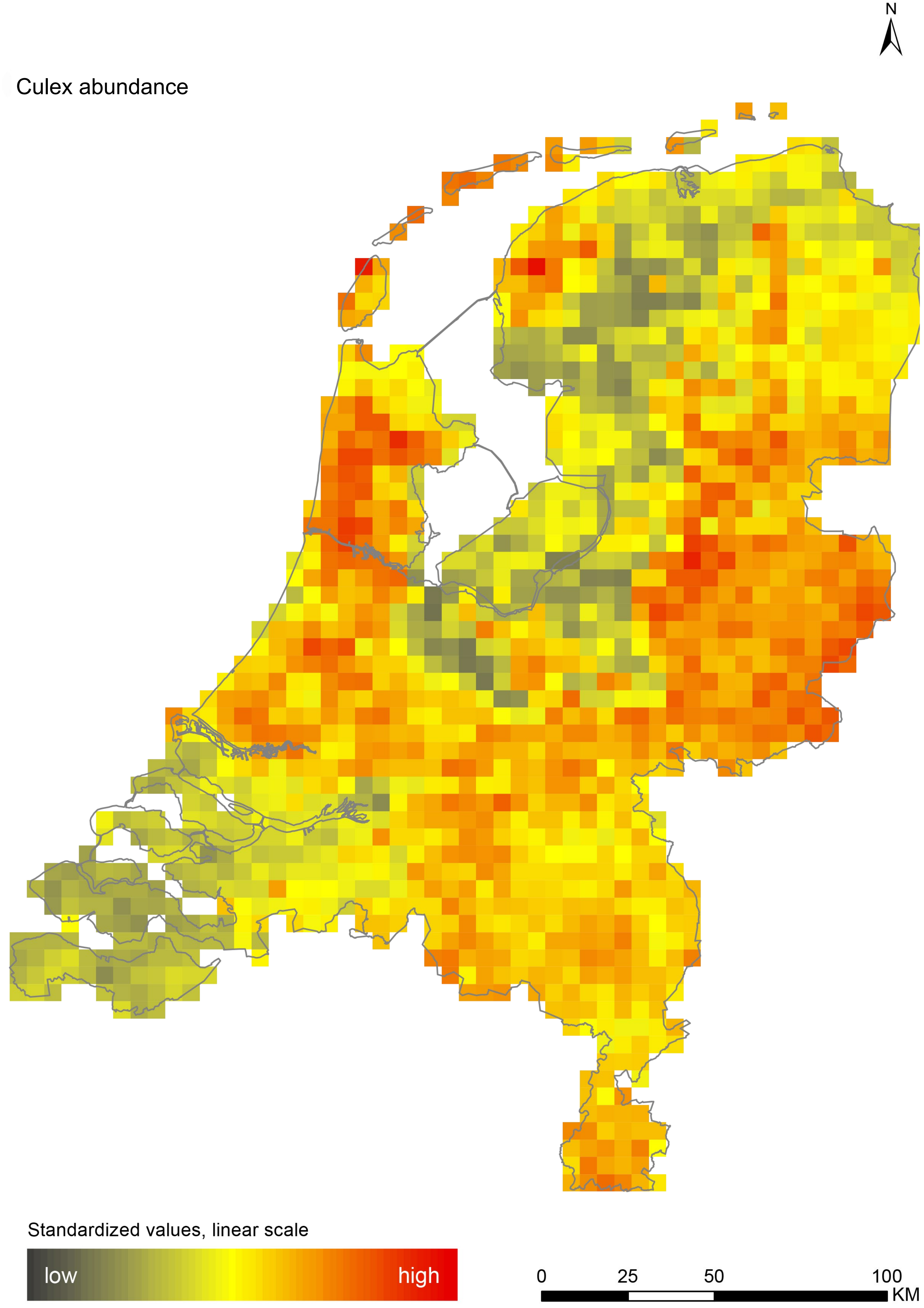
N
Culex abundance
Standardized values, linear scale
low
high
0
25
50
100
KM

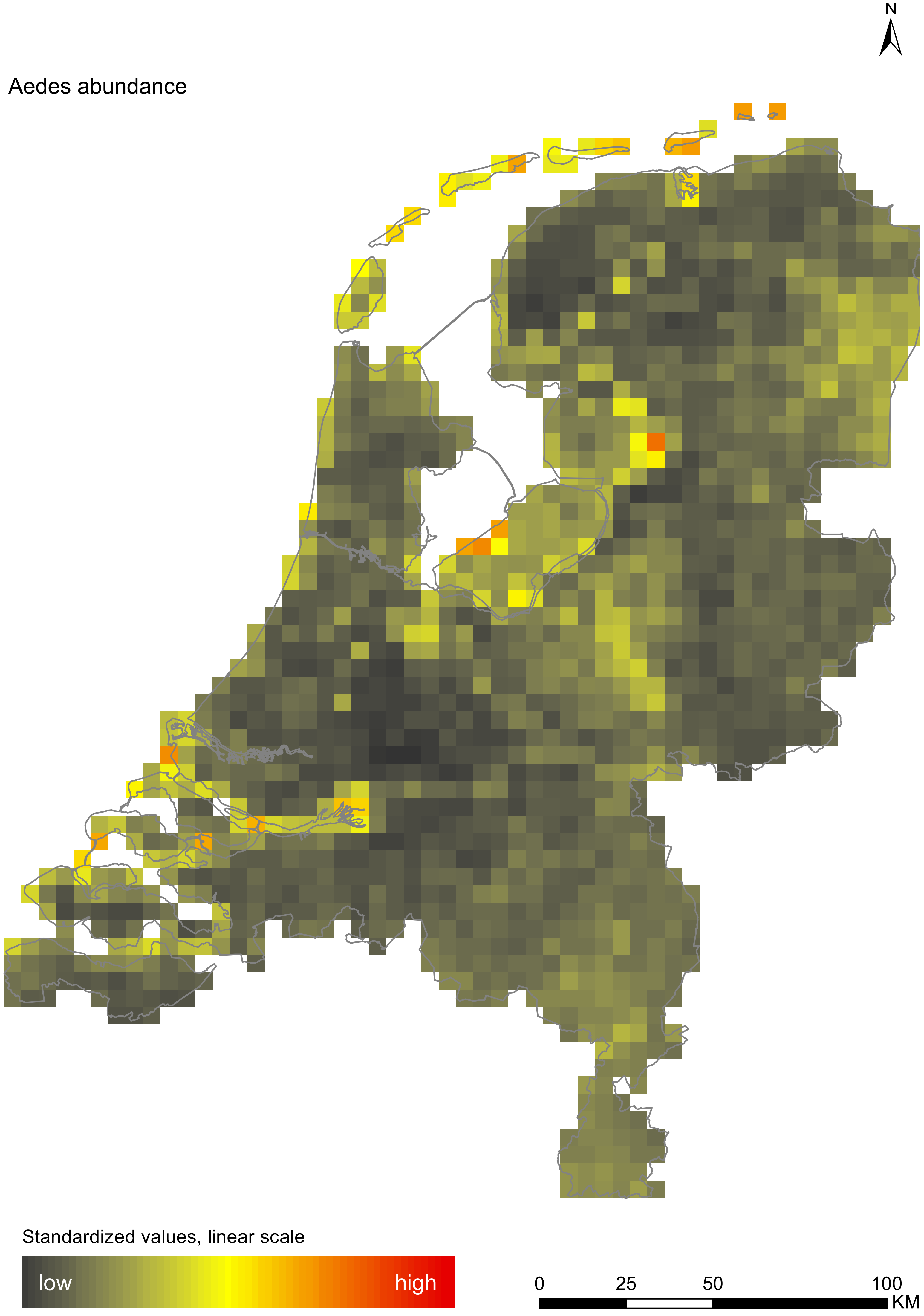
N
Aedes abundance
Standardized values, linear scale
low
high
0
25
50
100
KM

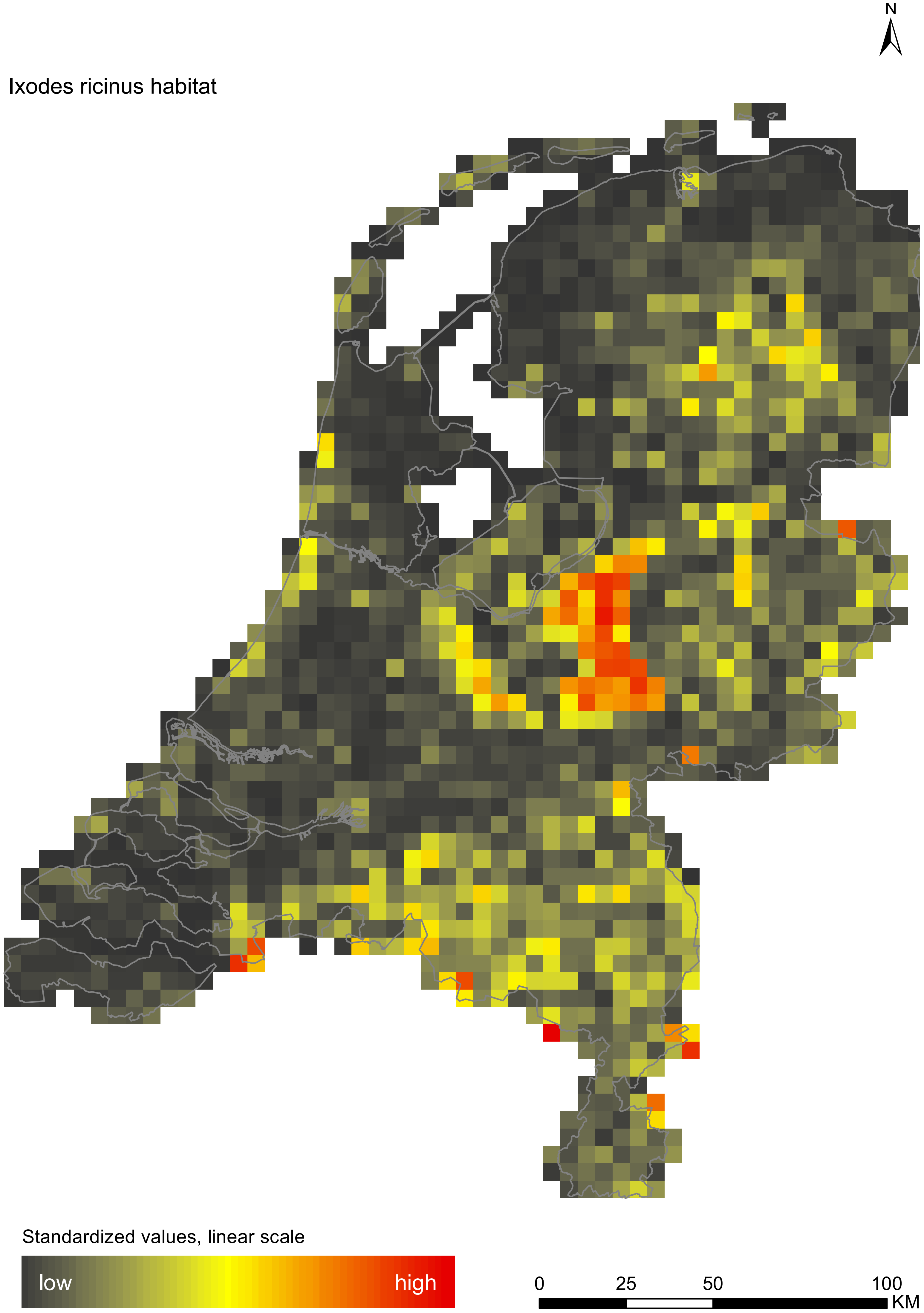
N
Ixodes ricinus habitat
Standardized values, linear scale
low
high
0
25
50
100
KM

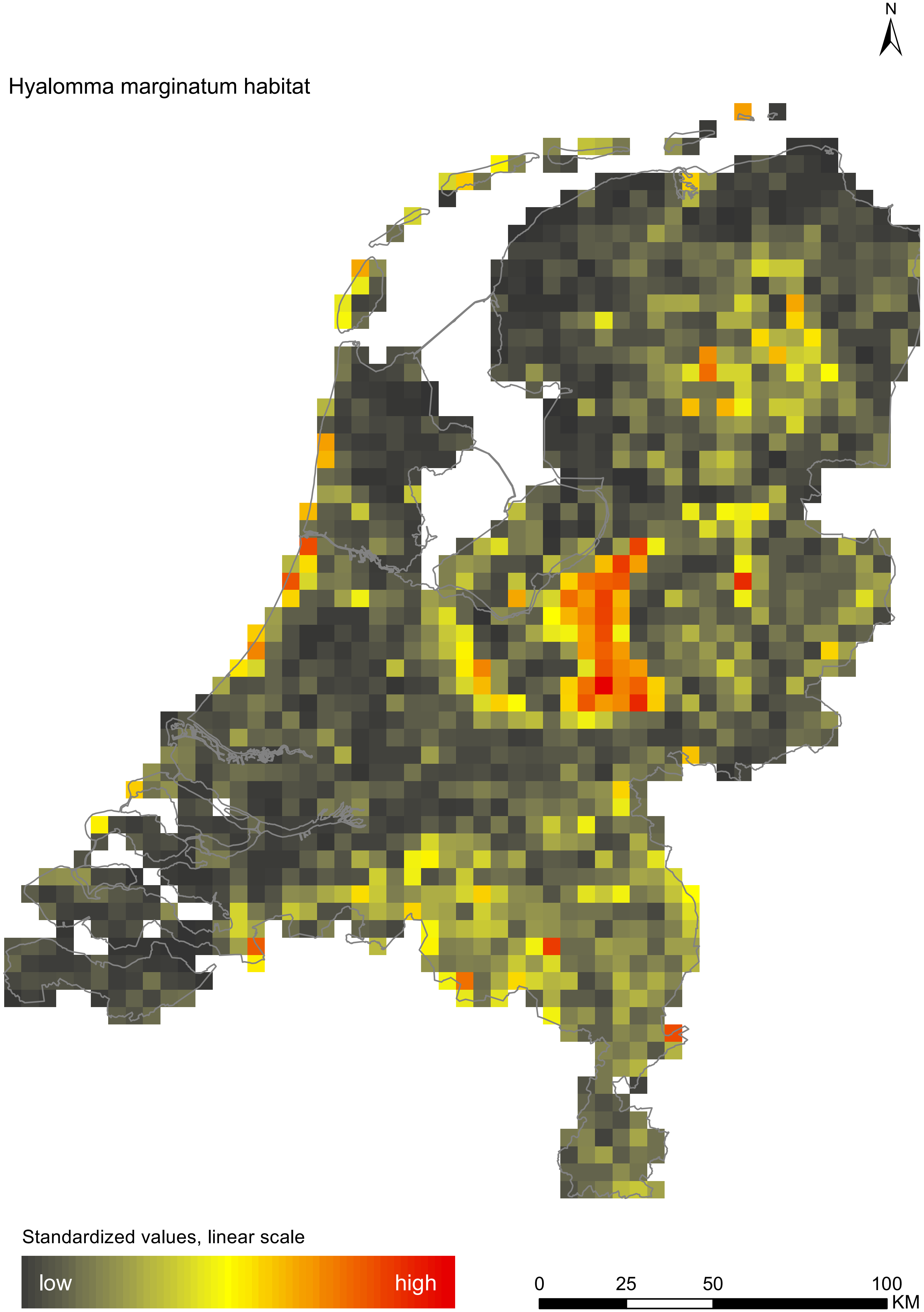
N
Hyalomma marginatum habitat
Standardized values, linear scale
low
high
0
25
50
100
KM

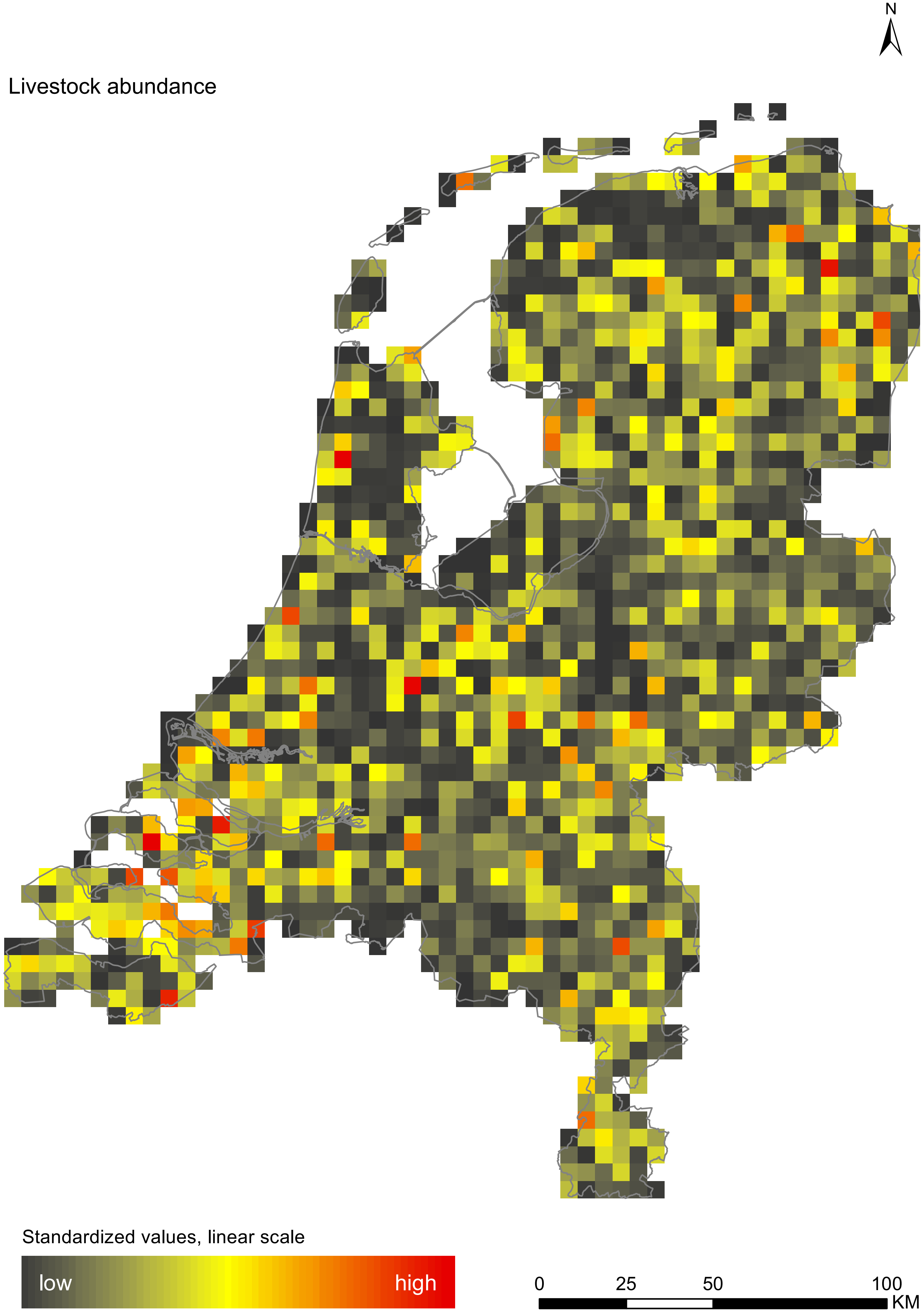
N
Livestock abundance
Standardized values, linear scale
low
high
0
25
50
100
KM

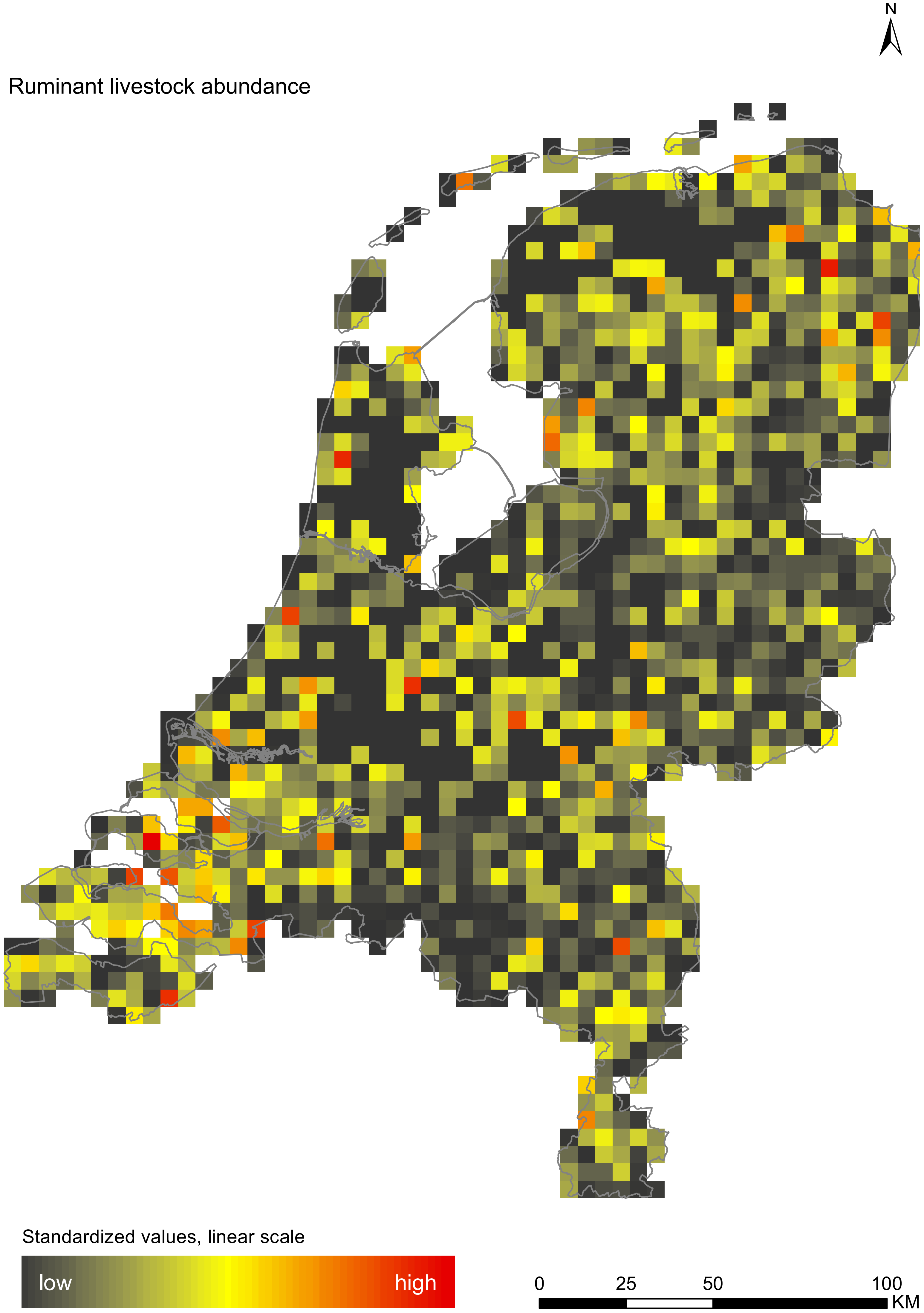
N
Ruminant livestock abundance
Standardized values, linear scale
low
high
0
25
50
100
KM

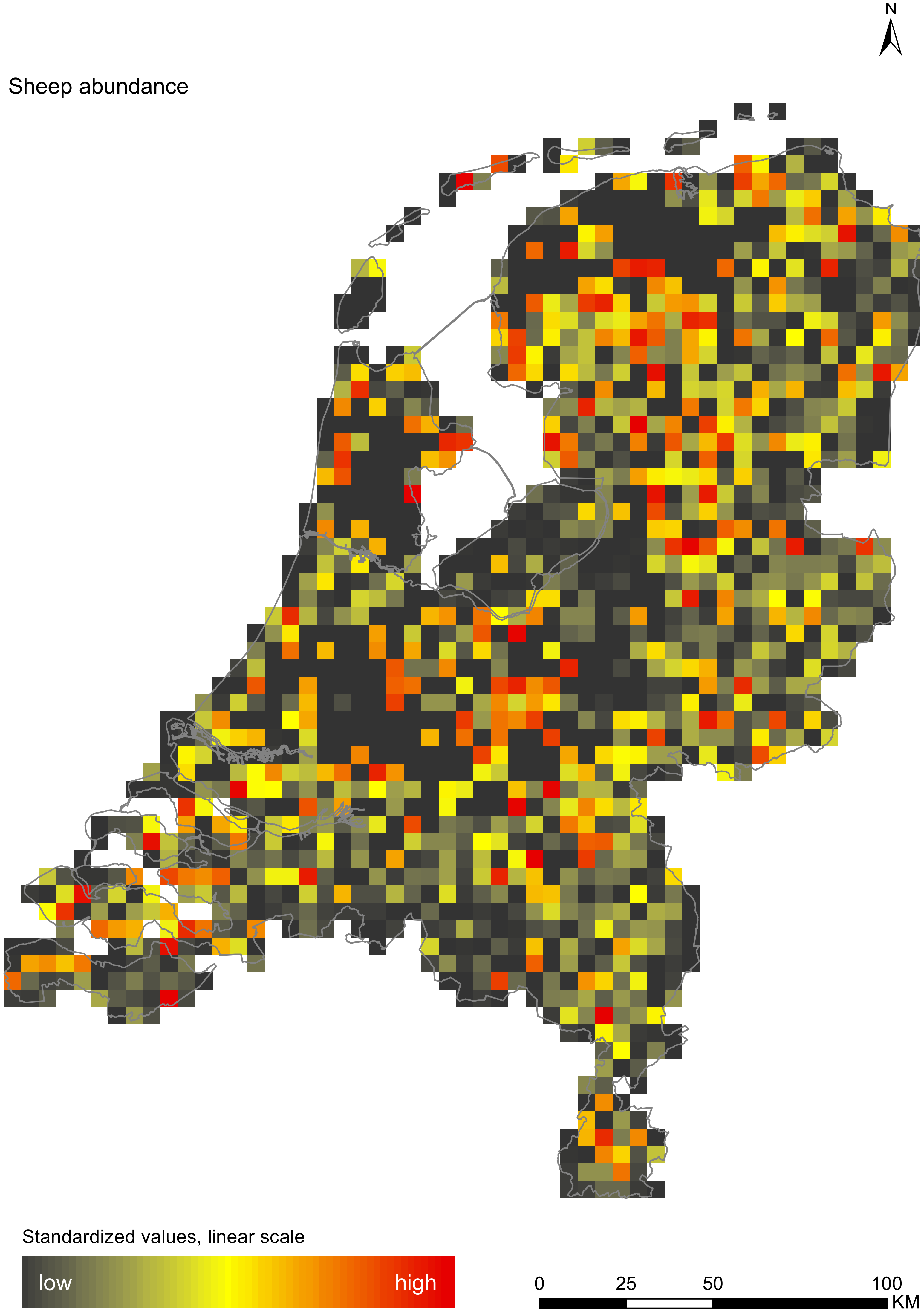
N
Sheep abundance
Standardized values, linear scale
low
high
0
25
50
100
KM

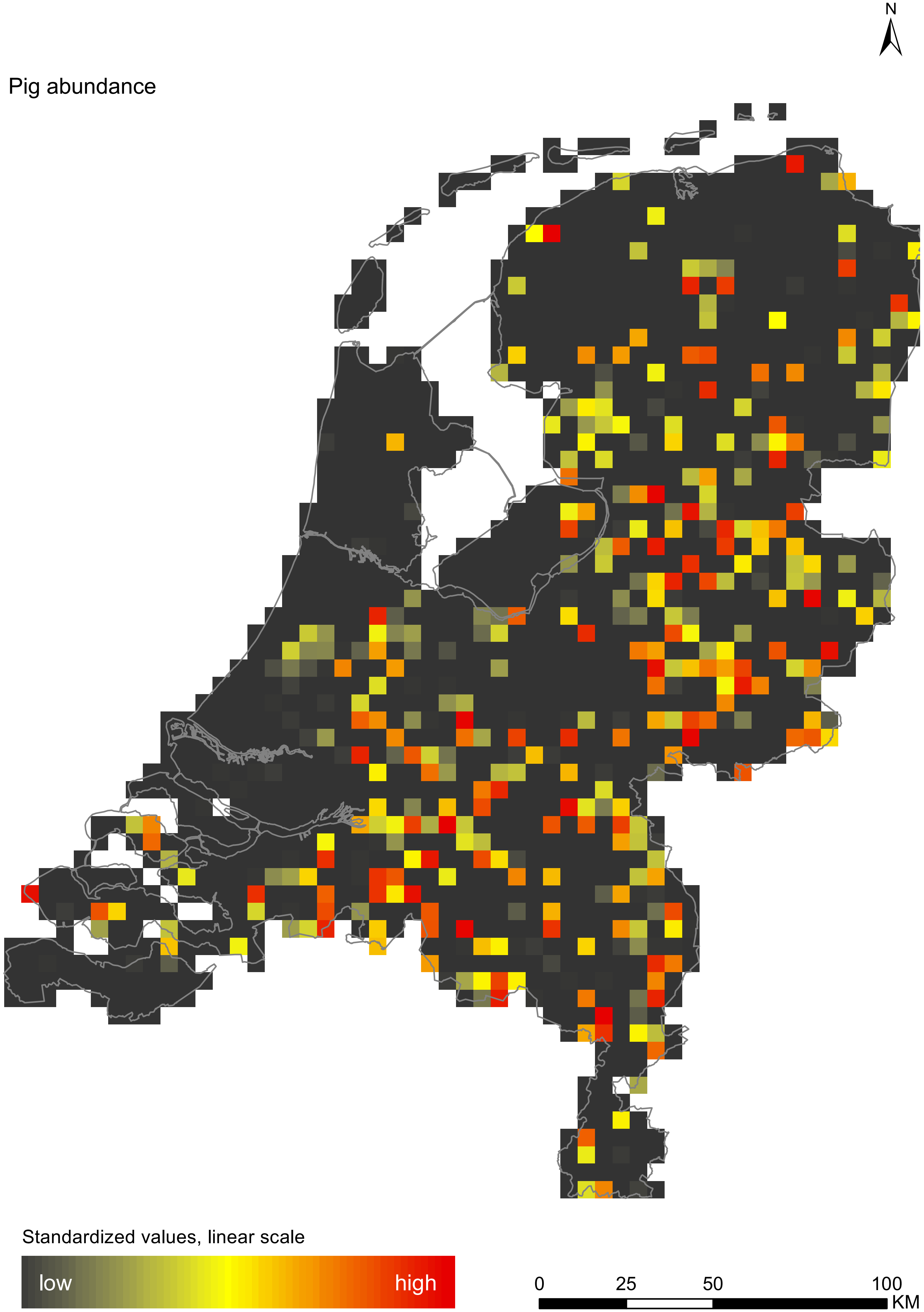
N
Pig abundance
Standardized values, linear scale
low
high
0
25
50
100
KM

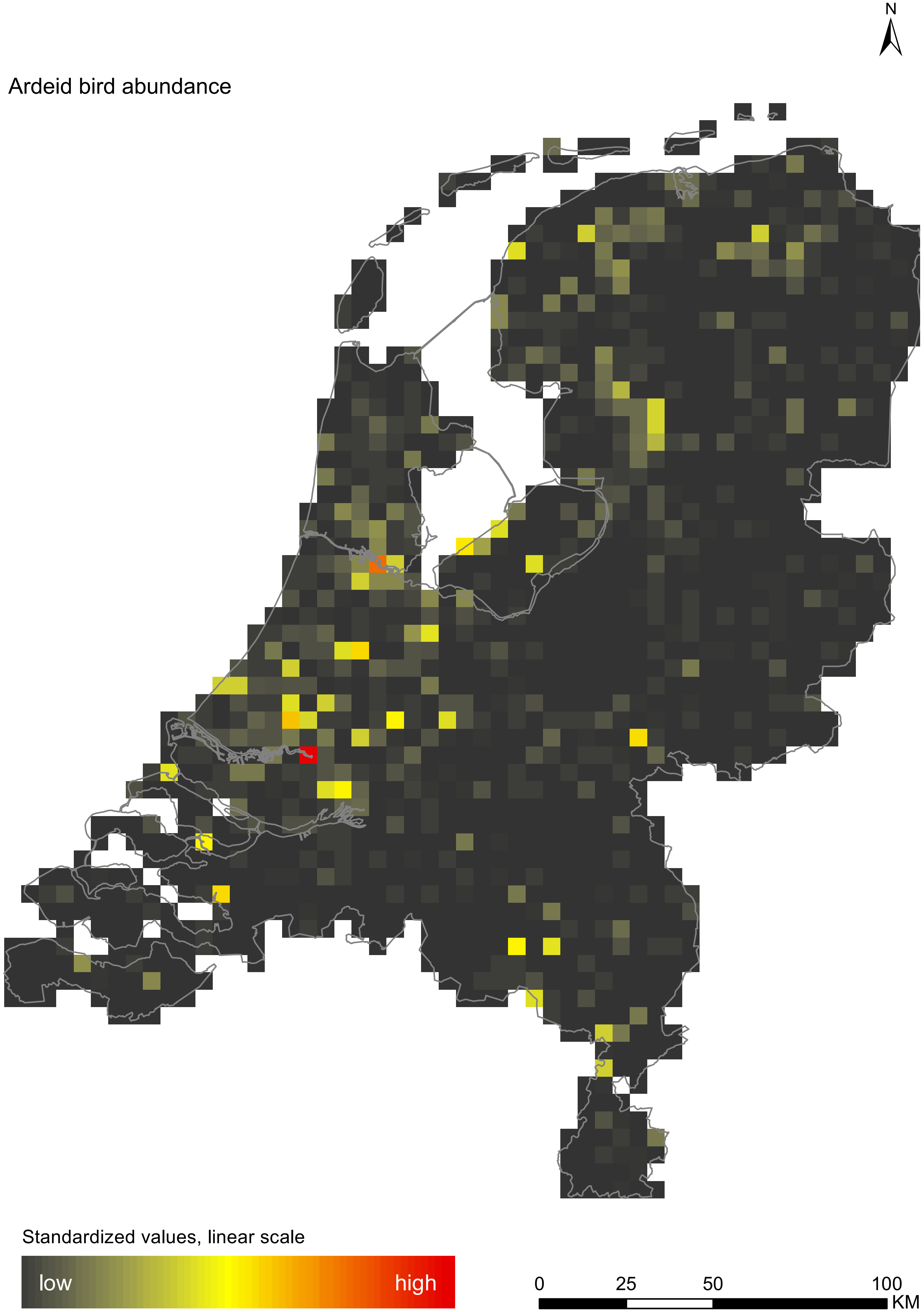

N
Ardeid bird abundance
Standardized values, linear scale
low
high
0
25
50
100
KM

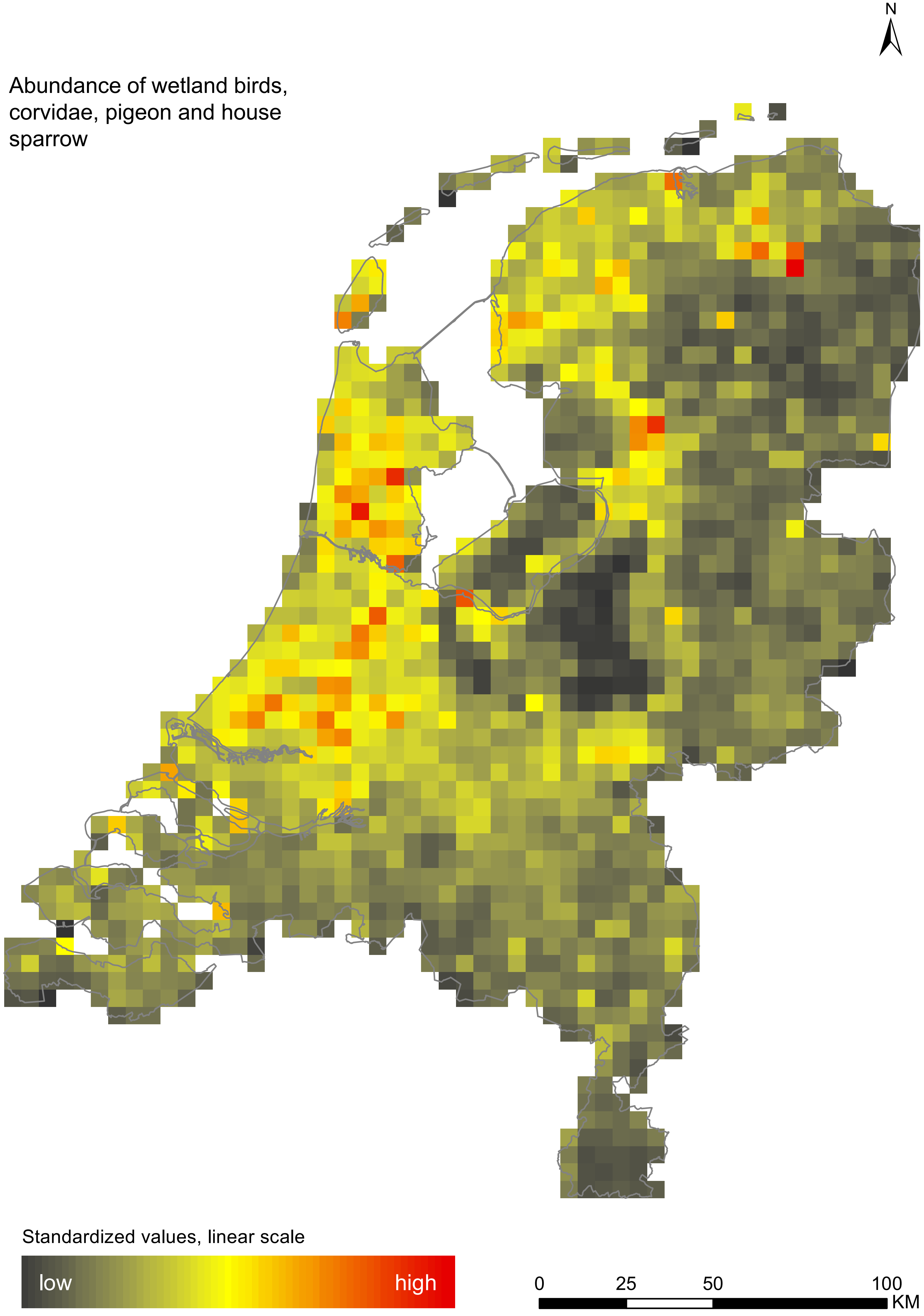
N
Abundance of wetland birds, corvidae, pigeon and house sparrow
Standardized values, linear scale
low
high
0
25
50
100
KM

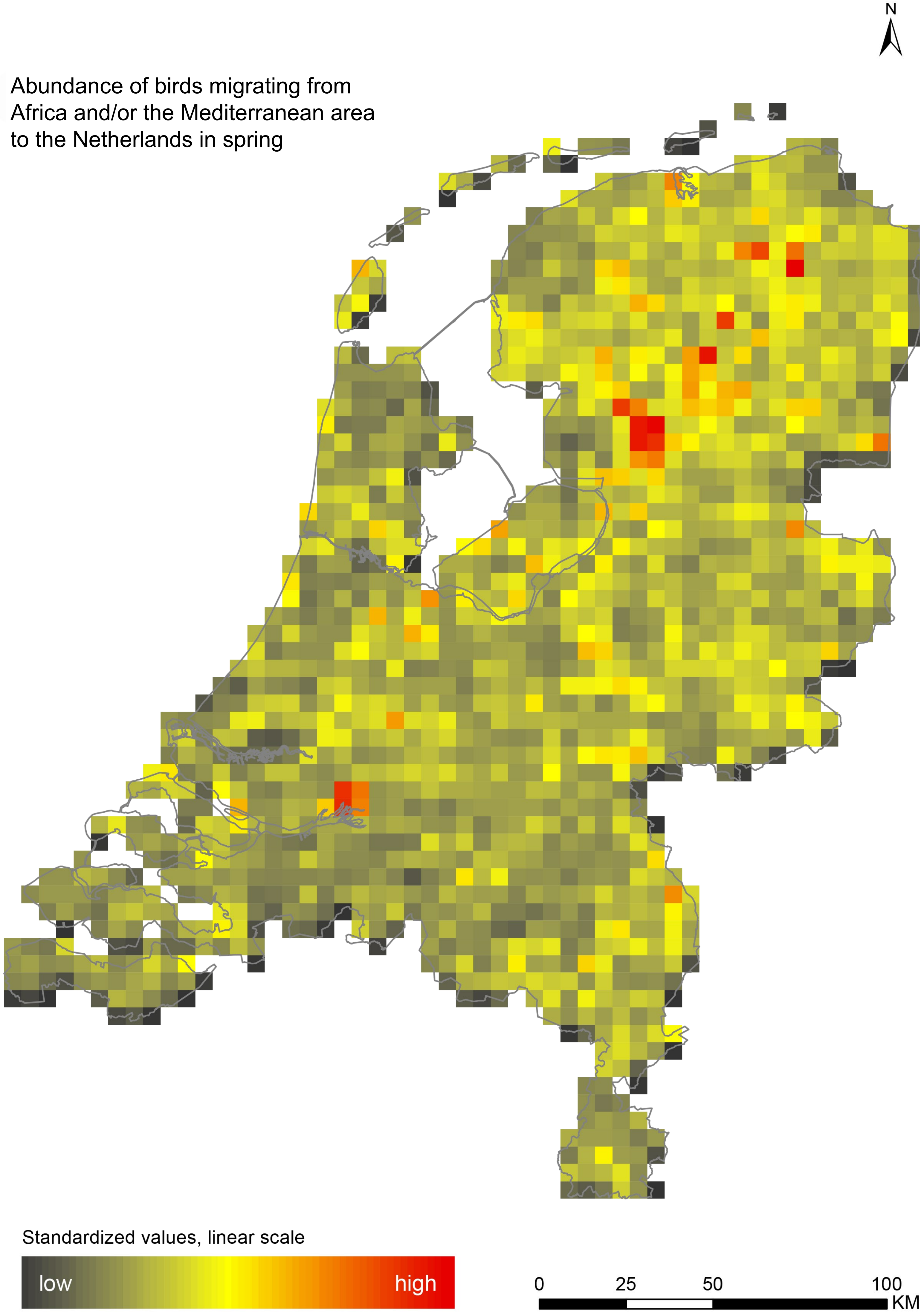
N
Abundance of birds migrating from
Africa and/or the Mediterranean area
to the Netherlands in spring
Standardized values, linear scale
low
high
0
25
50
100
KM

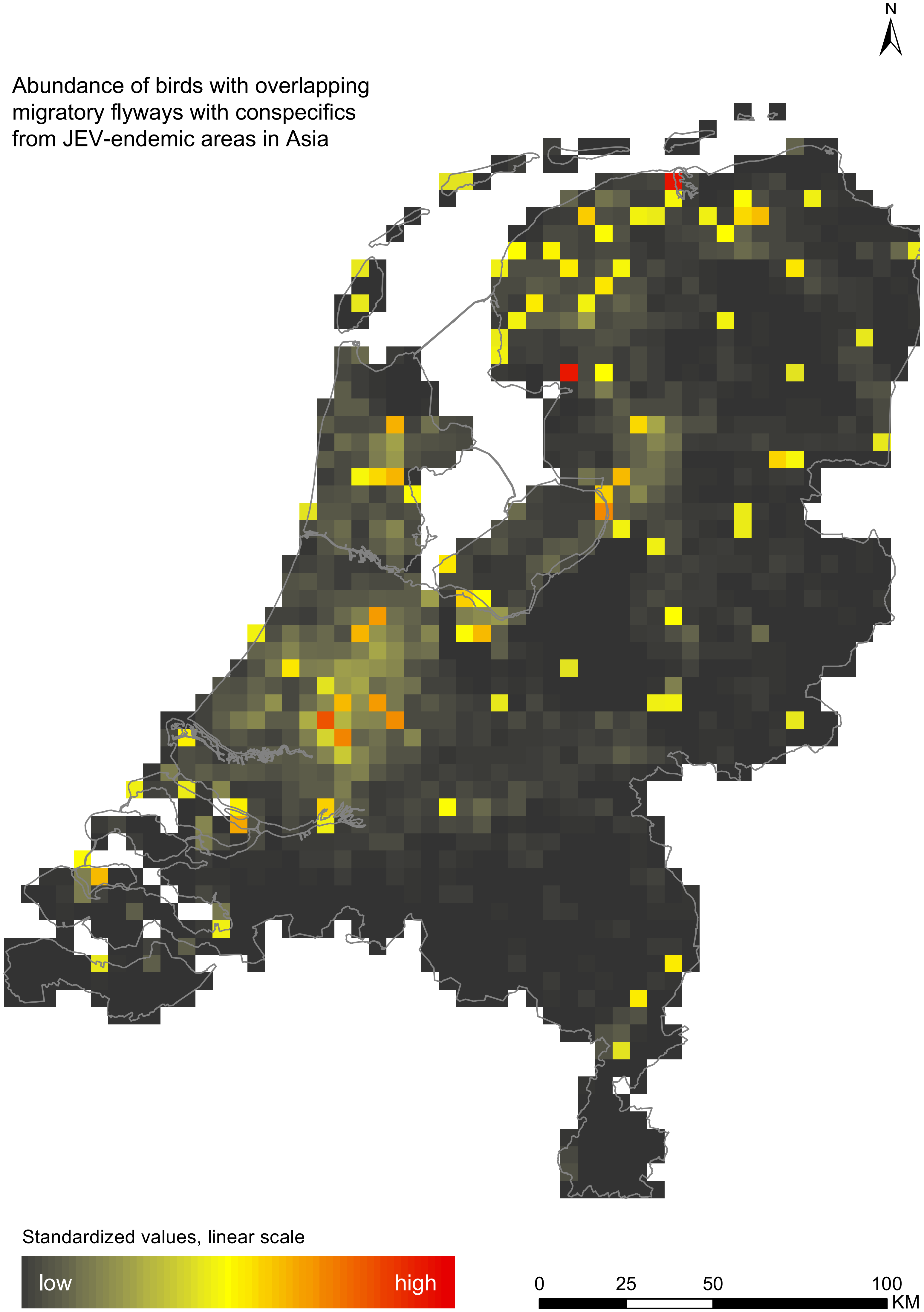
N
Abundance of birds with overlapping migratory flyways with conspecifics from JEV-endemic areas in Asia
Standardized values, linear scale
low
high
0
25
50
100
KM

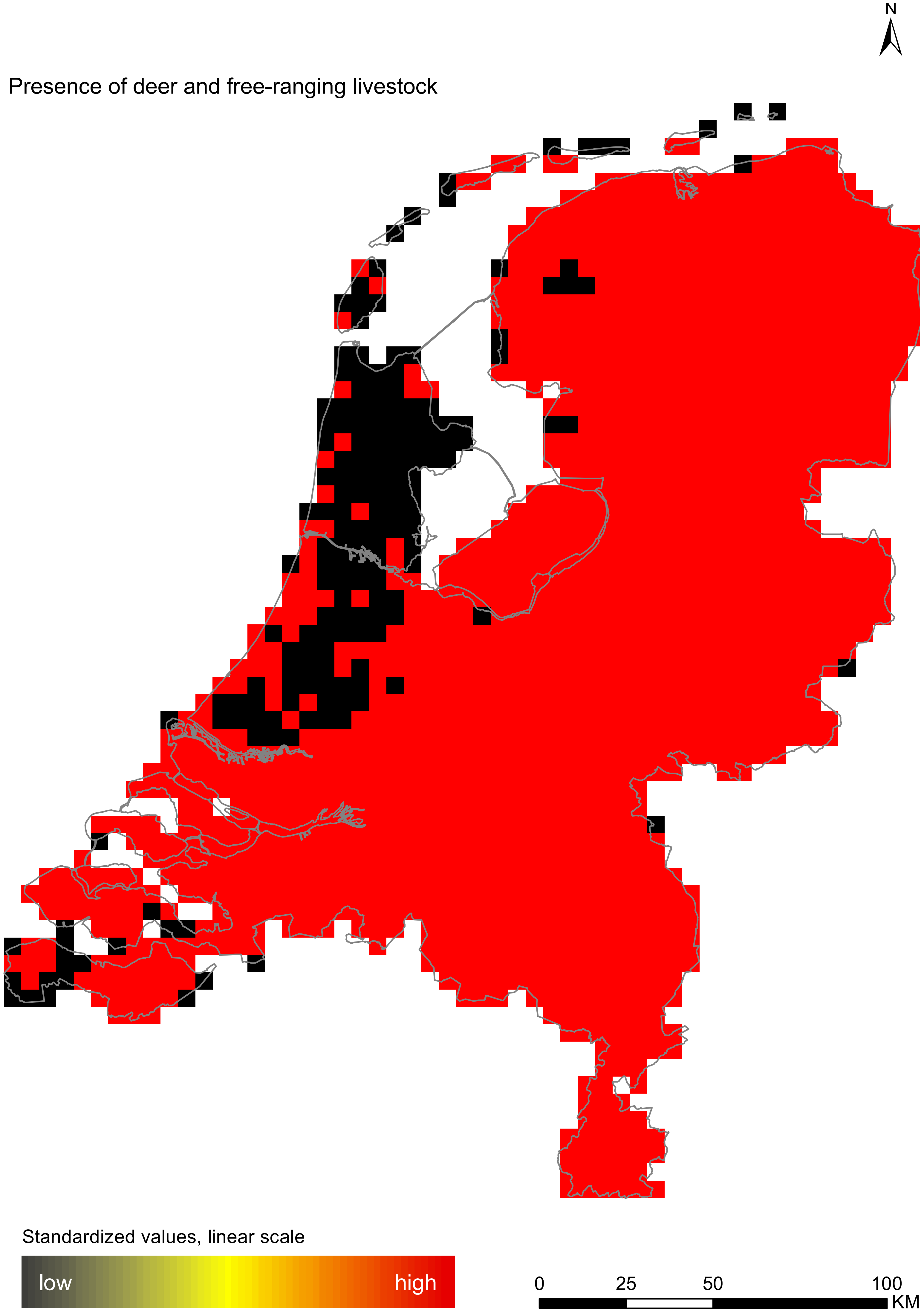
N
Presence of deer and free-ranging livestock
Standardized values, linear scale
low
high
0
25
50
100
KM
