# Supplementary material for: Spatial risk analysis for the introduction and circulation of six arboviruses in the Netherlands
Source: Parasit Vectors. 2020 Sep 10;13:464. doi: 10.1186/s13071-020-04339-0 (PMC7488554; doi:10.1186/s13071-020-04339-0)
Supplement: Supplementary file 4 — Additional file 4: Figures S20–S25. Schematic representation of the steps taken to construct the hazard maps for the establishment of each of the six arboviruses. [file 13071_2020_4339_MOESM4_ESM.pdf]

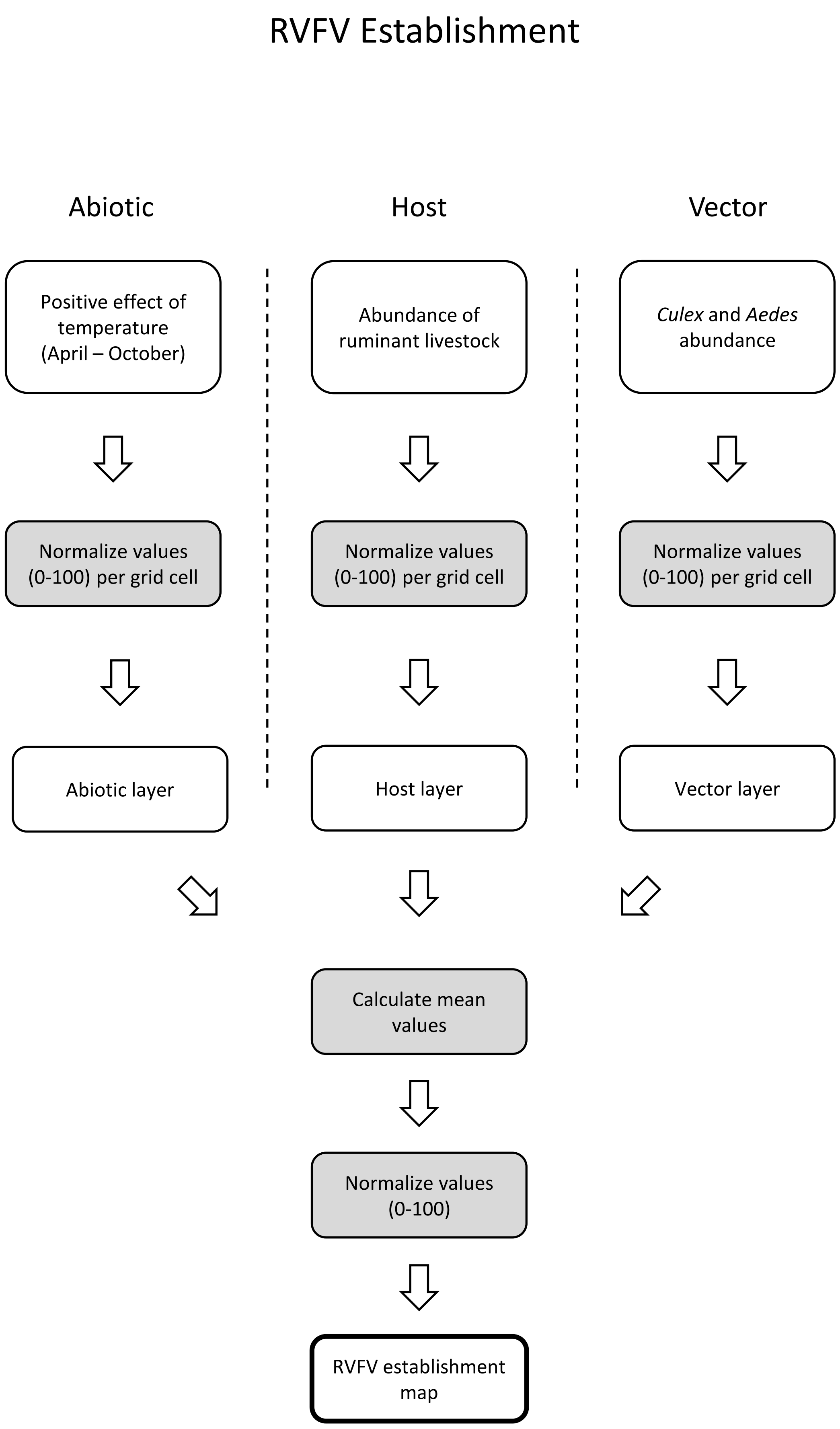
RVFV Establishment
Abiotic
Host
Vector
Positive effect of temperature (April – October)
Abundance of ruminant livestock
Culex and *Aedes* abundance
Normalize values (0-100) per grid cell
Normalize values (0-100) per grid cell
Normalize values (0-100) per grid cell
Abiotic layer
Host layer
Vector layer
Calculate mean values
Normalize values (0-100)
RVFV establishment map

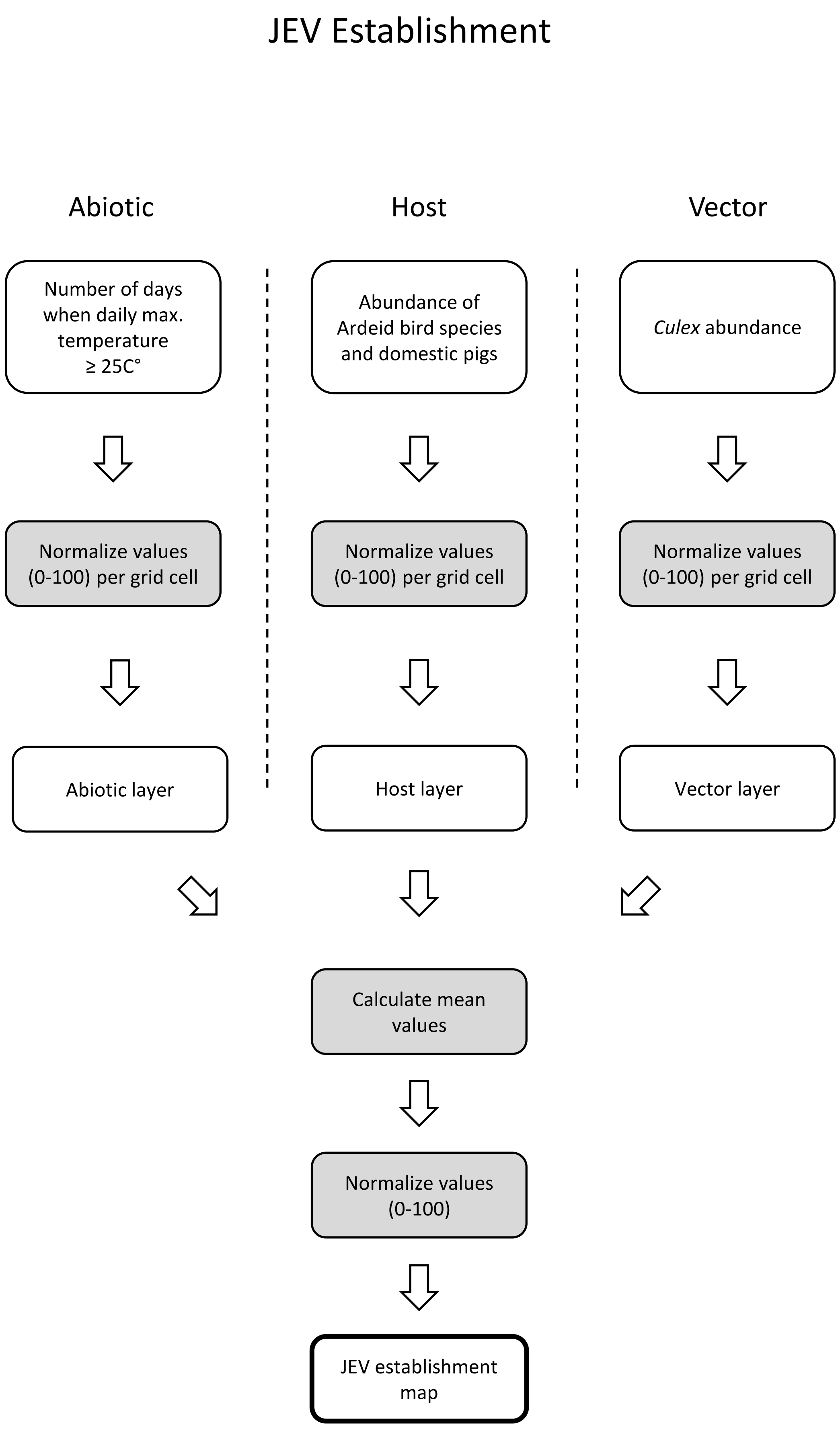

JEV Establishment
Abiotic
Host
Vector
Number of days when daily max. temperature ≥ 25C°
Abundance of Ardeid bird species and domestic pigs
Culex abundance
Normalize values (0-100) per grid cell
Normalize values (0-100) per grid cell
Normalize values (0-100) per grid cell
Abiotic layer
Host layer
Vector layer
Calculate mean values
Normalize values (0-100)
JEV establishment map

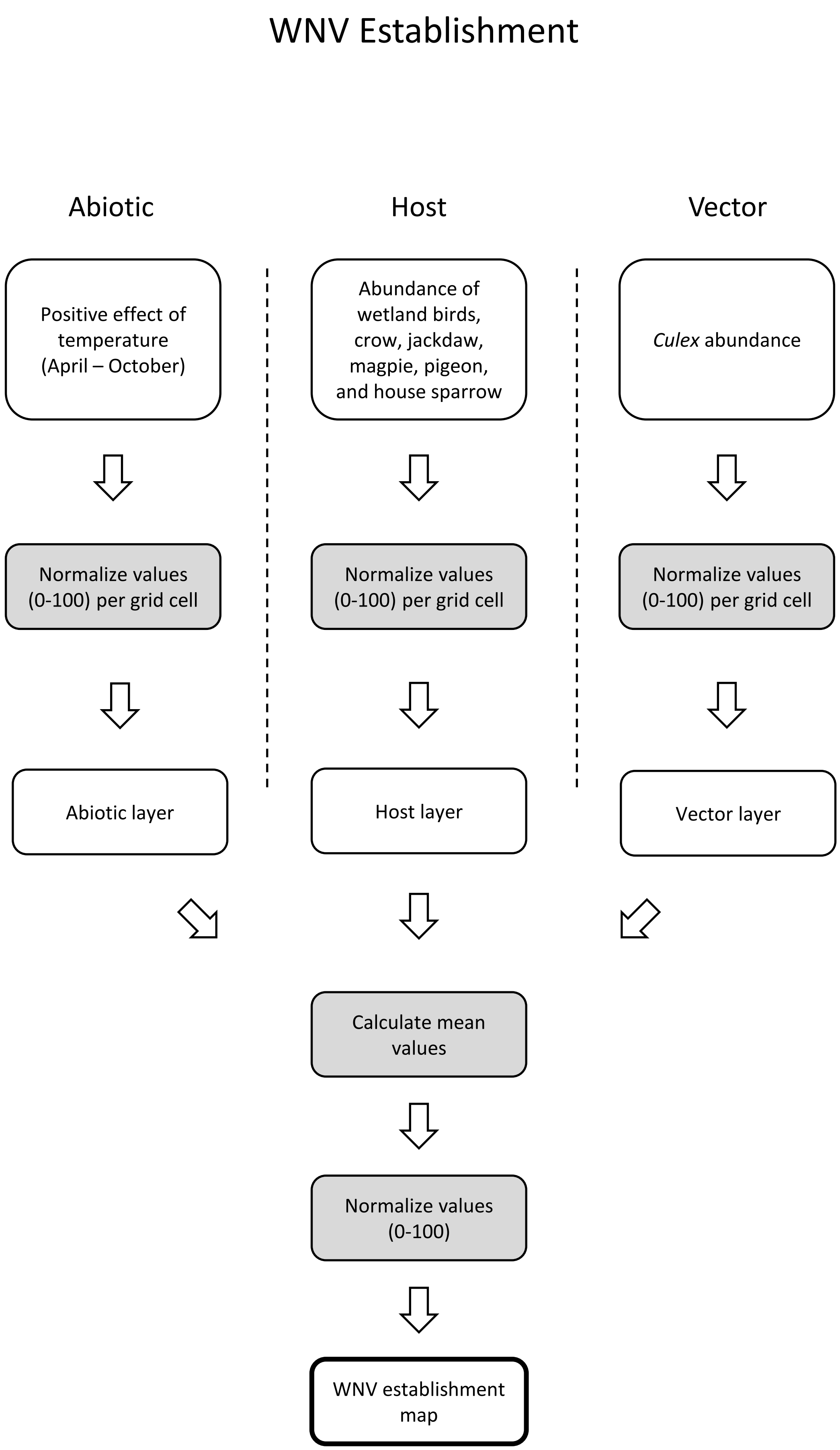

WNV Establishment
Abiotic
Host
Vector
Positive effect of temperature (April – October)
Abundance of wetland birds, crow, jackdaw, magpie, pigeon, and house sparrow
Culex abundance
Normalize values (0-100) per grid cell
Normalize values (0-100) per grid cell
Normalize values (0-100) per grid cell
Abiotic layer
Host layer
Vector layer
Calculate mean values
Normalize values (0-100)
WNV establishment map

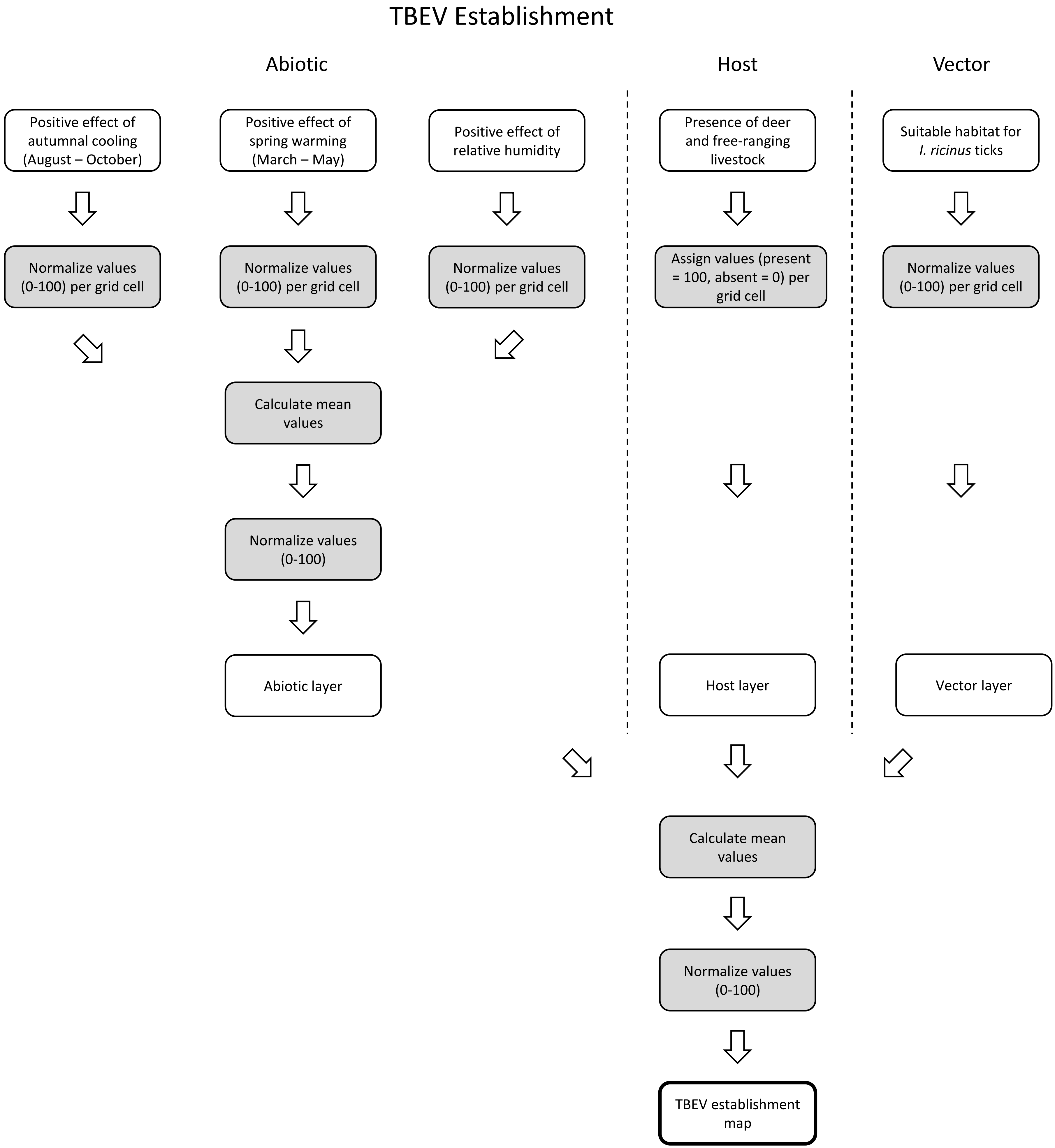
TBEV Establishment
Abiotic
Host
Vector
Positive effect of autumnal cooling (August – October)
Positive effect of spring warming (March – May)
Positive effect of relative humidity
Presence of deer and free-ranging livestock
Suitable habitat for I. ricinus ticks
Normalize values (0-100) per grid cell
Normalize values (0-100) per grid cell
Normalize values (0-100) per grid cell
Assign values (present = 100, absent = 0) per grid cell
Normalize values (0-100) per grid cell
Calculate mean values
Normalize values (0-100)
Abiotic layer
Host layer
Vector layer
Calculate mean values
Normalize values (0-100)
TBEV establishment map

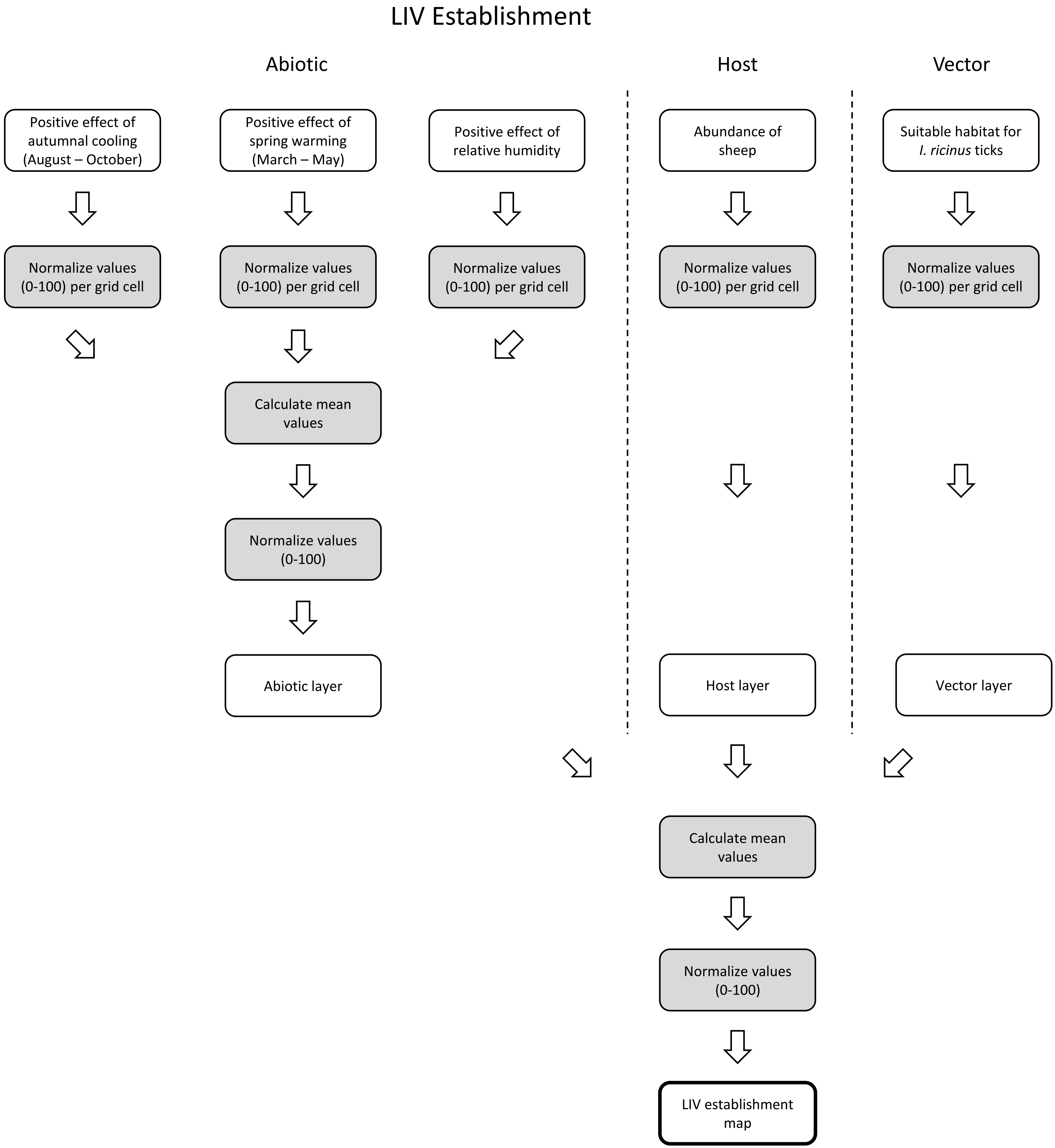
LIV Establishment
Abiotic
Host
Vector
Positive effect of autumnal cooling (August – October)
Positive effect of spring warming (March – May)
Positive effect of relative humidity
Abundance of sheep
Suitable habitat for I. ricinus ticks
Normalize values (0-100) per grid cell
Normalize values (0-100) per grid cell
Normalize values (0-100) per grid cell
Normalize values (0-100) per grid cell
Normalize values (0-100) per grid cell
Calculate mean values
Normalize values (0-100)
Abiotic layer
Host layer
Vector layer
Calculate mean values
Normalize values (0-100)
LIV establishment map

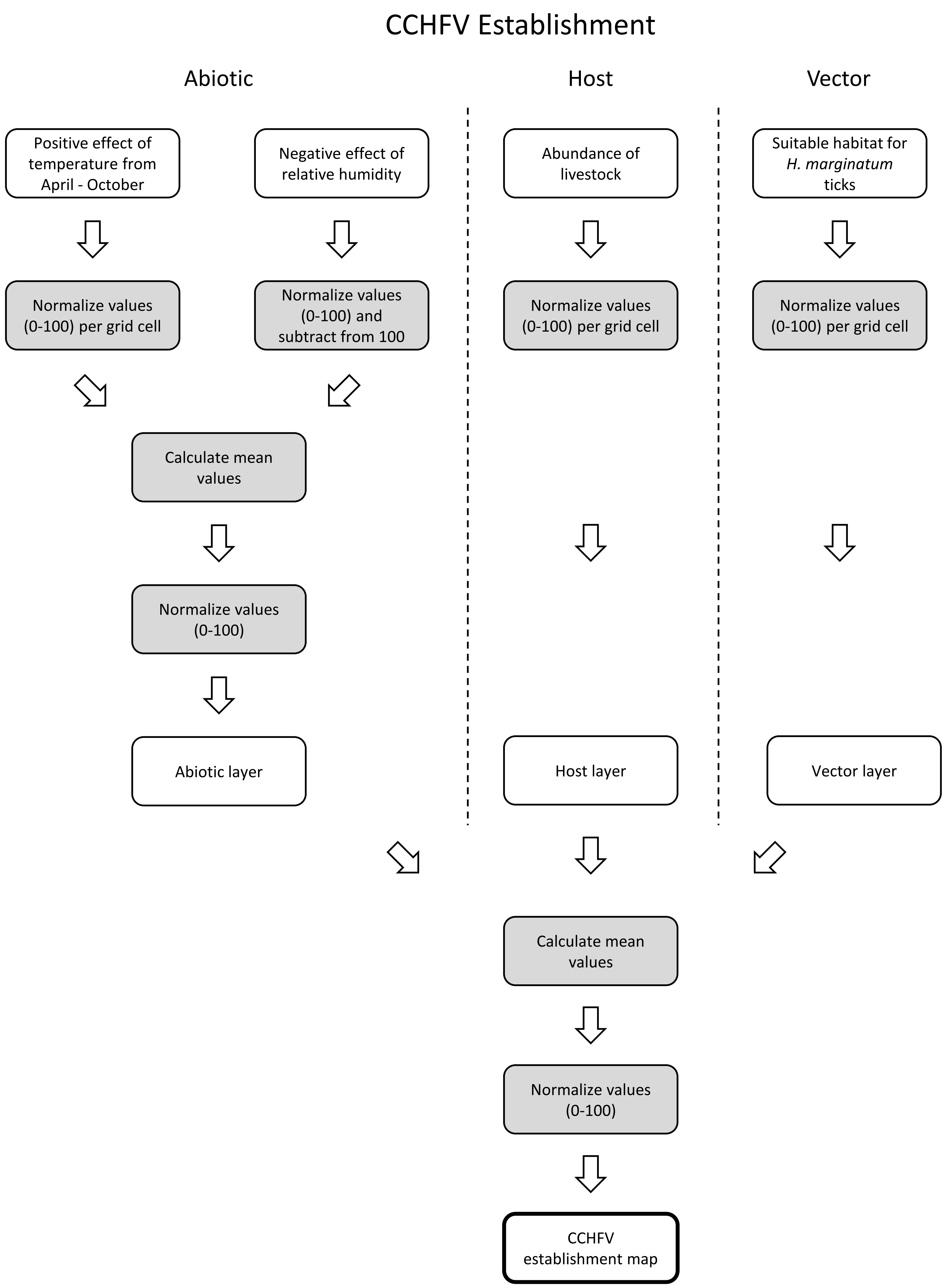
CCHFV Establishment
Abiotic
Host
Vector
Positive effect of temperature from April - October
Negative effect of relative humidity
Abundance of livestock
Suitable habitat for *H. marginatum* ticks
Normalize values (0-100) per grid cell
Normalize values (0-100) and subtract from 100
Normalize values (0-100) per grid cell
Normalize values (0-100) per grid cell
Calculate mean values
Normalize values (0-100)
Abiotic layer
Host layer
Vector layer
Calculate mean values
Normalize values (0-100)
CCHFV establishment map
